# Design and Synthesis of Novel Antimicrobial Acyclic and Heterocyclic Dyes and Their Precursors for Dyeing and/or Textile Finishing Based on 2-*N*-Acylamino-4,5,6,7-tetrahydro-benzo[*b*]thiophene Systems

**DOI:** 10.3390/molecules16086271

**Published:** 2011-07-26

**Authors:** Hoda Zaki Shams, Rafat Milad Mohareb, Maher Helmy Helal, Amira El-Sayed Mahmoud

**Affiliations:** 1 Department of Chemistry, Faculty of Science, Helwan University, Ain Helwan, Cairo, P.O. 11790, Egypt; 2 Department of Organic Chemistry, Faculty of Pharmacy, October University for Modern Sciences and Arts, October City, P.O. 12613, Egypt; 3 Department of Chemistry, Faculty of Science, Cairo University, Giza, P.O. 12311, Egypt

**Keywords:** heterocyclic, dyes, antimicrobial activity, textile finish, fastness

## Abstract

A series of novel polyfunctionalized acyclic and heterocyclic dye precursors and their respective azo (hydrazone) counterpart dyes and dye precursors based on conjugate enaminones and/or enaminonitrile moieties were synthesized. The dyes and their precursors are based on 2-cyano-*N*-(3-cyano-4,5,6,7-tetrahydrobenzo[*b*]thiophen-2-yl)-acetamide, 2-ethoxycarbonyl-*N*-(3-cyano-4,5,6,7-tetrahydrobenzo[*b*]thiophen-2-yl)-acetamide or 2-phenylcarbamoyl-*N*-(3-cyano-4,5,6,7-tetrahydrobenzo[*b*]thiophen-2-yl)-acetamide systems as precursors. The latter compounds were used to synthesize polyfunctional thiophene-, thiazole-, pyrazole, pyridine-, pyrimidine-, oxazine-, as well as acyclic moieties. The dyes and dye precursors were characterized by elemental analysis and spectral methods. All dyes and their precursors were screened *in vitro* and evaluated for both their antibacterial and antifungal activities. MIC data of the novel dye systems and their respective precursors showed significant antimicrobial activity against most tested organisms. Some compounds exhibited comparable or even higher efficiency than selected standards. Dyes were applied at 5% depth for disperse dyeing of nylon, acetate and polyester fabrics. Their spectral characteristics and fastness properties were measured and evaluated.

## 1. Introduction

The growing interest in heterocyclic azo dye chemistry is focused on designing new synthetic approaches to these materials, theoretical calculations, and applications in various industrial fields. Besides having important-applications as textile colorants [1,2,3,4,5,6,7,8], they find increasing accessibility in photo-responsive biomaterials [9], optical sensing of metal ions [10,11,12], non-linear optics (NLO) and photoelectronics [11,13,14,15,16]. Functional dyes with special finishing capabilities is currently an area of active research [17]. Finishes for textile materials such as water repellent dyes [18,19], anti-UV radiation dyes [20,21,22], and antimicrobial dyes [23,24] have been discussed.

Many researchers have explored in the field of simultaneous dyeing and functional finishing of textiles, such as the simultaneous dyeing and durable press finishing of cotton [25,26,27], the combination of dyeing and durable press finishing of silk [28,29], the simultaneous dyeing and finishing of wool [30], as well as the simultaneous dyeing and antimicrobial finishing of acrylic fabrics [31]. Other reports concerning antimicrobial functional finishing of synthetic fabrics *via* treatment with either *N*-haloamine moieties or by quaternary ammonium salts (QAS) has been discussed [32,33]. Both treatments may limit or affect the dyeing of the finished fabric. Chlorine bleach is needed in *N*-haloamine treatments, while the use of QAS may occupy some available dye sites within the fabrics, thus interfering with the dyeing behavior of the resultant fabric.

At the other extreme enaminone systems have been reported as important building blocks in heterocyclic synthesis [34,35]. Azo dyes based on the conjugate enaminones or enaminonitriles containing the respective conjugate systems O=C-(C=C)_n_-N- and N≡C-(C=C)_n_-N- have attracted ongoing attention. Besides having important dyeing capabilities, they provide the basis for the expanding field of structural studies [36,37,38]. Their importance as azo dyes and azo pigments depends on their donating-attracting effects which leads to their existence in several tautomeric forms connected with different types of hydrogen bonds. The existence of azo-hydrazone tautomerism affects the basic characteristics (colour tone, photostability) of azo dyes which can be used for the design of compounds having required colour properties. 

Based upon the above considerations and in a continuation of our program directed toward the synthesis of bioactive heterocyclic systems [39,40,41,42], we therefore consider it worthwhile to design and synthesize heterocyclic functional azo dyes that combine the favorable properties of conjugate enaminone, enaminonitrile and azo systems with the hope that both dyeing and antimicrobial capabilities for textile finish may be achieved.

The synthetic strategy of the investigated dyes and their precursors depended on the competition of the reaction pathways which followed nucleophilic displacement, β-attack, Gewald type reaction, dinucleophilic bielectrophilic attack, dipolar cyclization and condensation reactions. This led to the diversity of the reaction products.

The novel compounds could be leads for the development of new functional materials with special finish properties for textile fabrics. Moreover, the results of the present study may point that the novel products could be useful as synthetic precursors for azo- and azomethine ligands or polymethine dyes which may be suitable for both electronic and optical applications.

## 2. Results and Discussion

### 2.1. Chemistry

**Scheme 1:**
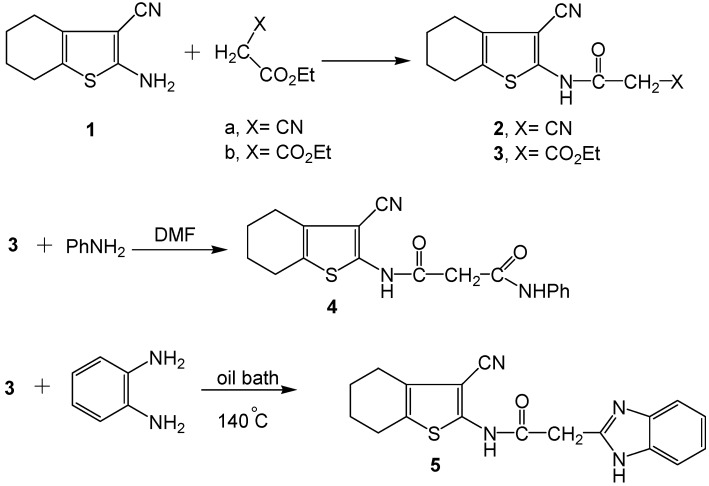
Synthesis of precursors **2**, **3**, **4** and **5**.

**Scheme 2:**
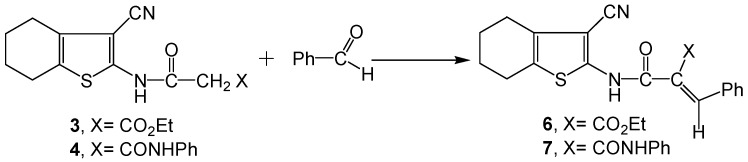
Synthesis of benzylidene derivatives **6** and **7**.

**Scheme 3:**
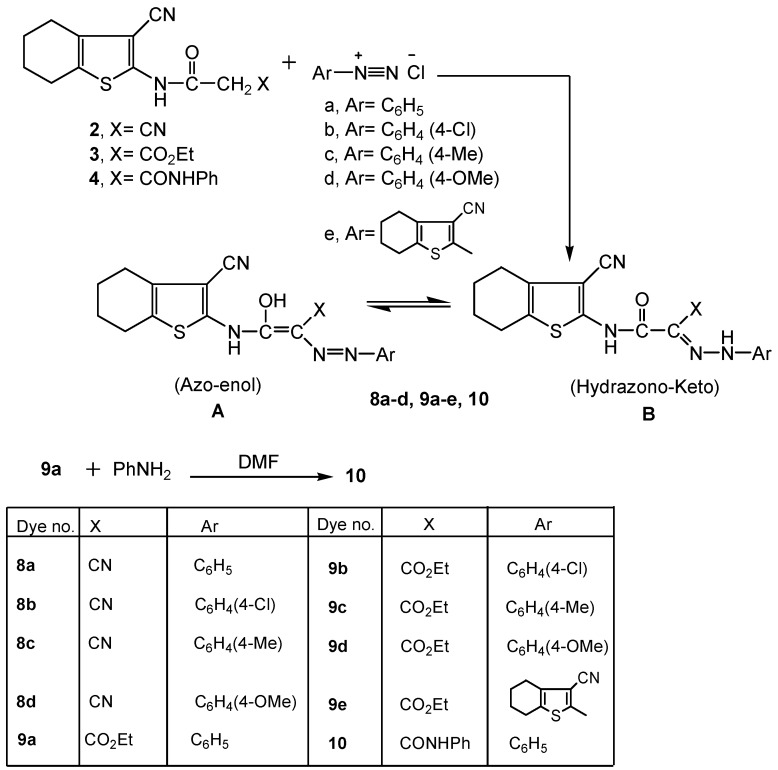
Synthesis of acyclic aryl azo (hydrazone) dyes **8a-d**, **9a-e** and **10**.

**Scheme 4:**
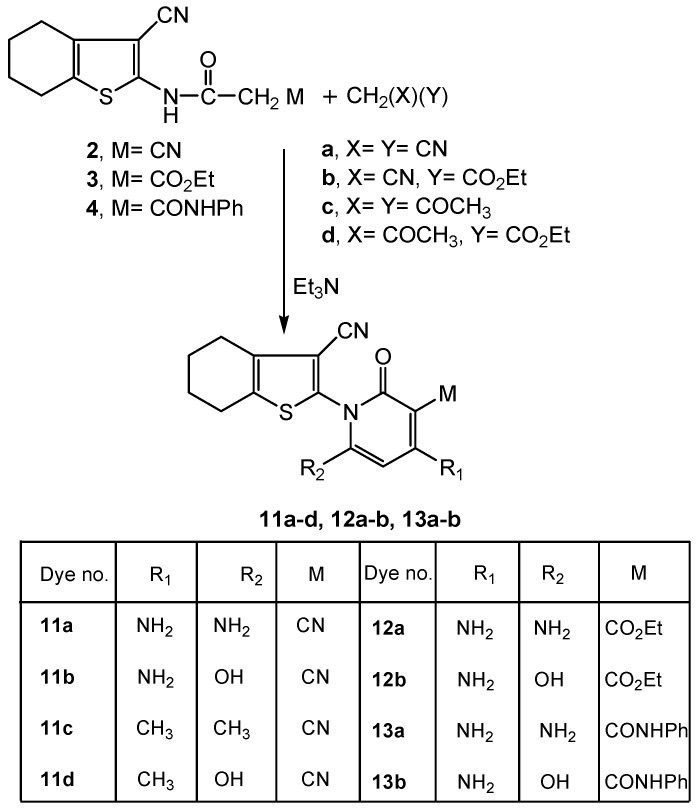
Synthesis of functionalized 2-oxopyridine dye precursors **11a-d**, **12a-b** and **13a-b**.

**Scheme 5:**
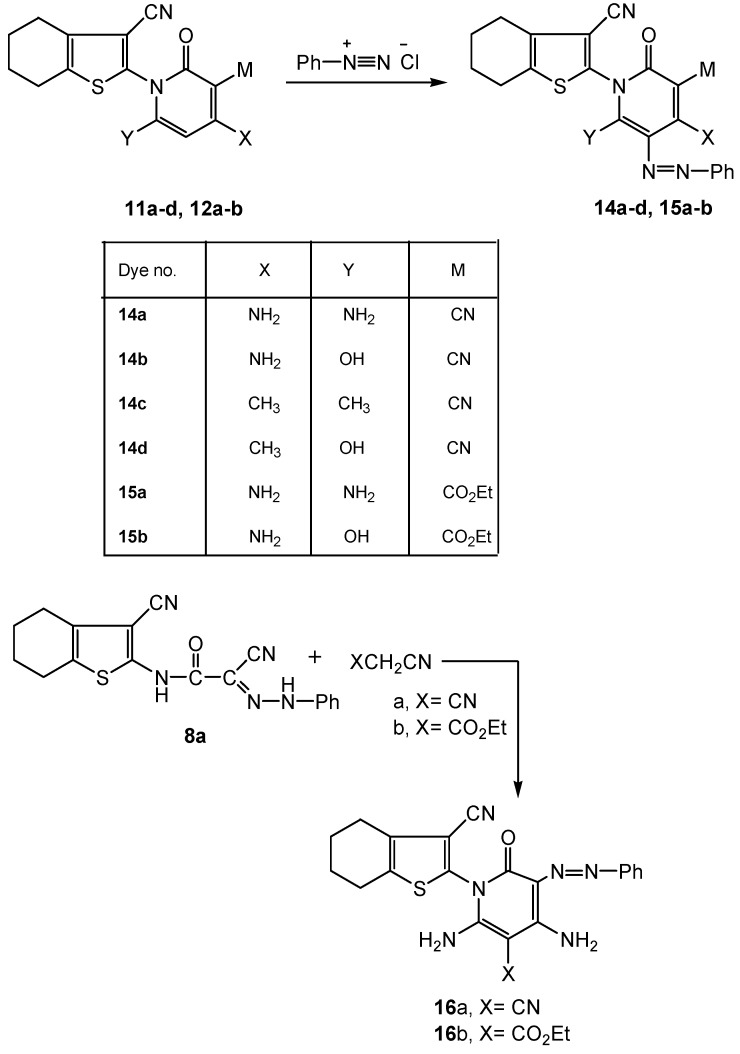
Synthesis of functionalized 2-oxopyridine azo (hydrazone) dyes **14a-d**, **15a-b** and **16a-b**.

**Scheme 6:**
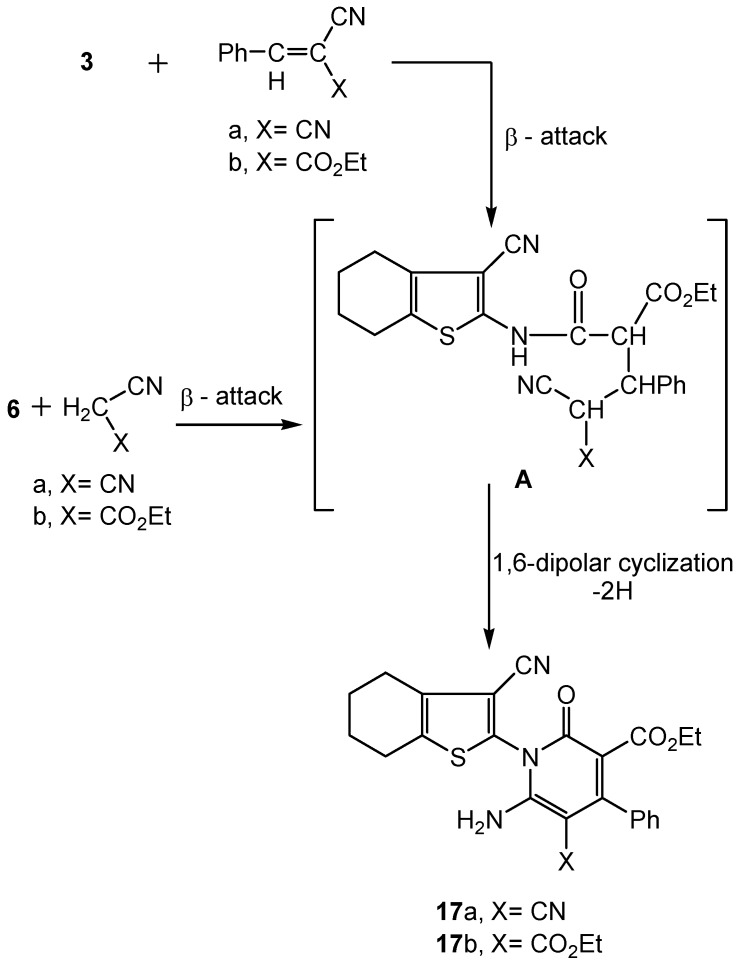
Synthesis of functionalized 2-oxopyridine derivatives **17a-b**.

**Scheme 7:**
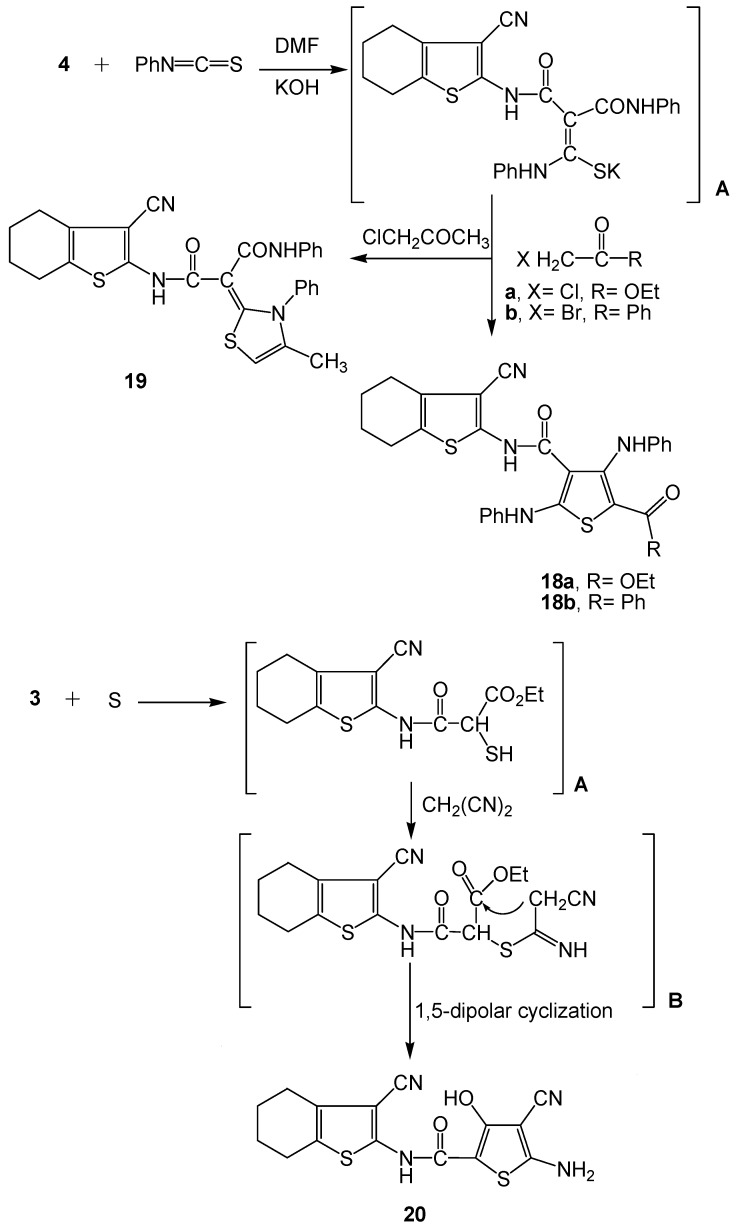
Synthesis of functionalized thiophenes **18a-b** and **20** and thiazole derivative **19**.

**Scheme 8:**
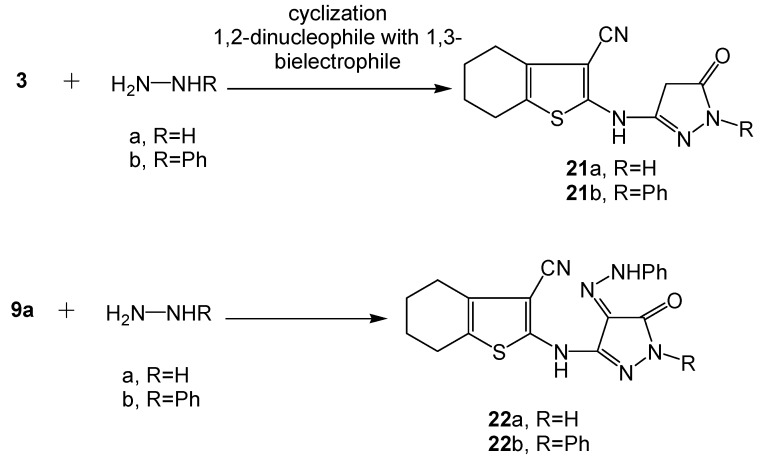
Synthesis of functionalized oxopyrazole dye precursors **21a-b **and respective dyes **22a-b**.

**Scheme 9:**
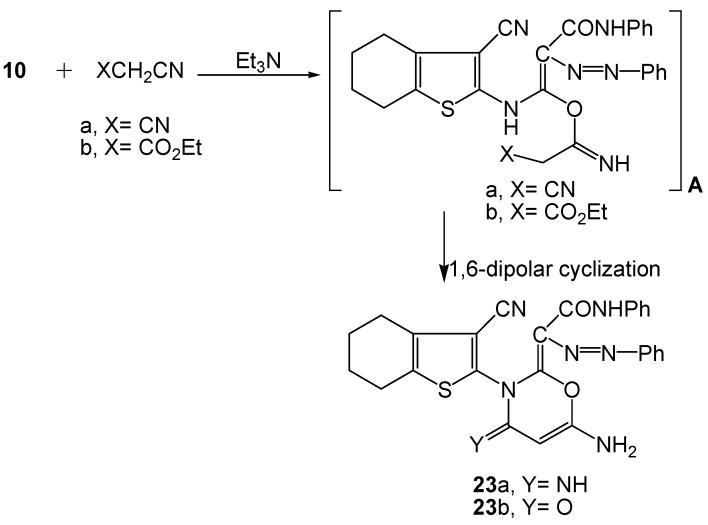
Synthesis of functionalized oxazinylidene azo dyes **23a-b**.

**Scheme 10:**
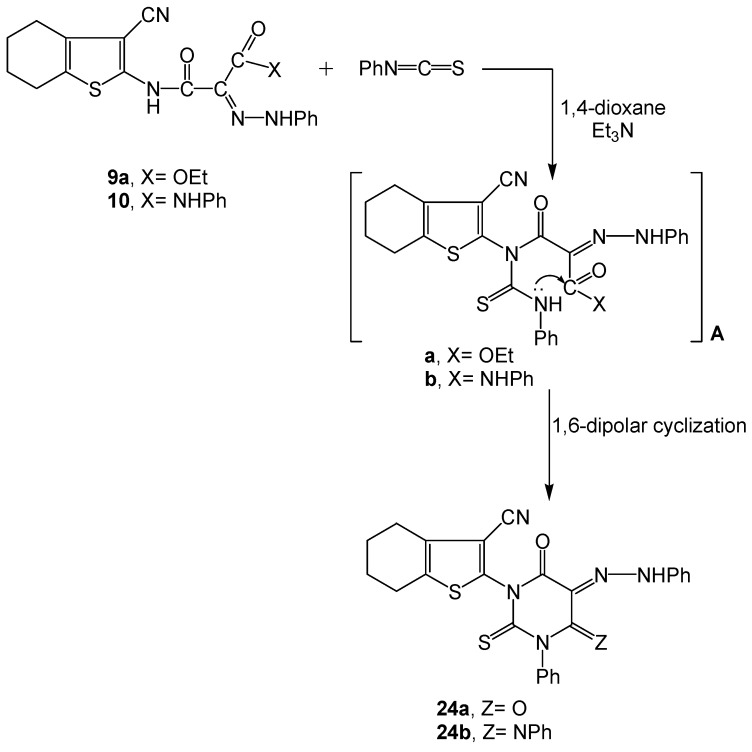
Synthesis of functionalized 2-oxopyrimidine phenyl hydrazone dyes **24a-b**.

**Scheme 11:**
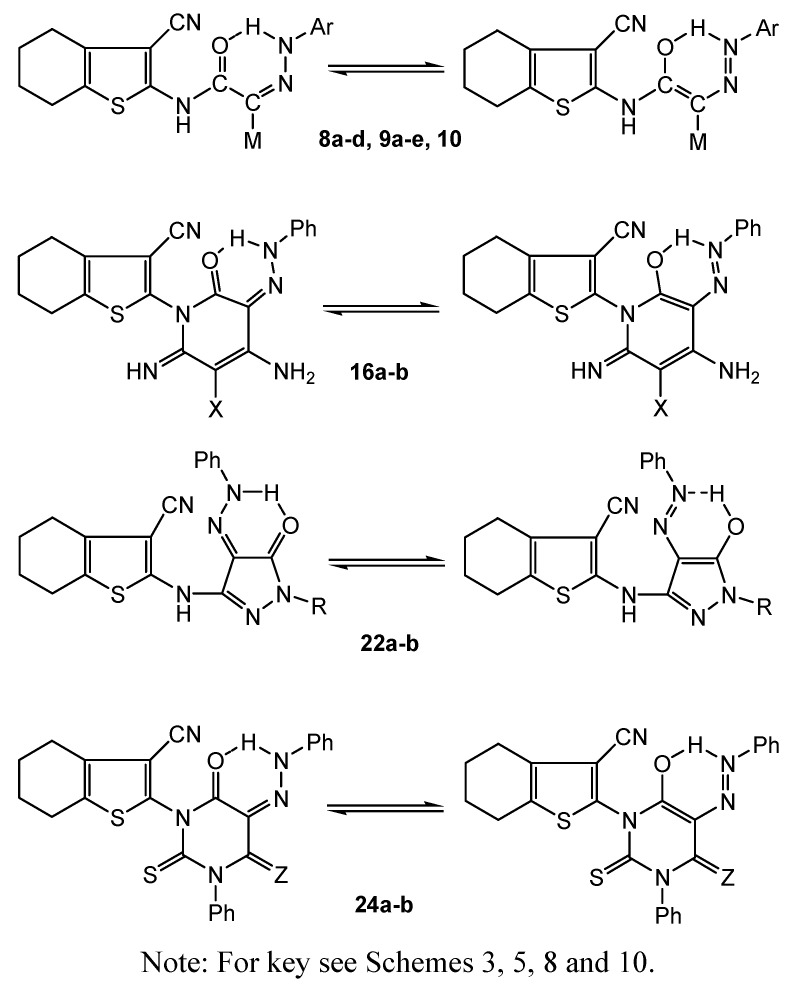
Possible azo ligand structure of some synthesized dyes *via* intramolecular chelation by H-bond.

In the present work, we report the synthetic strategies for preparation of a series of novel functionalized acyclic and heterocyclic azo (hydrazone) dyes and dye precursors comprising conjugate enaminones and/or enaminonitrile moieties. The synthesized systems are based on three synthetic key precursors, namely, 2-cyano-*N*-(3-cyano-4,5,6,7-tetrahydrobenzo[*b*]thiophen-2-yl)-acetamide (**2**) [42], *N*-(3-cyano-4,5,6,7-tetrahydrobenzo[*b*]thiophen-2-yl)-malonamic acid ethyl ester (**3**) or *N*-(3-cyano-4,5,6,7-tetrahydrobenzo[*b*]thiophen-2-yl)-*N^/^*-phenyl-malonamide (**4**). The mechanistic pathways for our protocolS are outlined in SchemeS 1-11. Micro-analytical data, infrared (IR), ^1^H-Nuclear Magnetic Resonance (^1^H-NMR), ^13^C-NMR and mass spectral (MS) data are indicated in the Experimental section. The key precursors **2** and **3** [50] were obtained *via* the reaction of 2-amino-3-cyano-4,5,6,7-tetrahydrobenzo[*b*]thiophene (**1)** [43] with the respective active methylene reagents (XCH_2_CO_2_Et; X=CN; X=CO_2_Et). When **3** was reacted with aniline or 1,2-phenylenediamine, the corresponding phenylmalonamide or benzoimidazolyl acetamide derivatives **4** and **5** were obtained Scheme 1. 

The reaction involved nucleophilic displacement (compound **4**) or 1,4-dinucleophilic cyclization of the diamine reagent with ethoxycarbonyl bielectrophile (compound **5**). The data obtained from analytical and spectral studies are fully consistent with the proposed structures **3-5** (Experimental section). Common features of compounds **3-5** were their strong amide C=O absorptions around 1652 cm^−1^ (IR), δ-^1^H multiplets about δ 1.72-2.93 ppm that integrated for four cyclohexene CH_2_ protons, δ-^1^H singlets about δ 4.04 ppm of the acetamido CH_2_ protons, as well as δ-^1^H singlets around δ 8.37-11.81 ppm (D_2_O exchangeable) due to the acetamido NH proton (^1^H-NMR). Compound **3** revealed, in its ^1^H-NMR spectrum, a triplet at δ 1.20 ppm and a quartet at δ 3.38 ppm due to its ester CH_3_ and CH_2_ protons, respectively. Mass spectra of compounds **3-5** displayed [M^+^] ion peaks at *m/z* 292, 339 and 336, respectively. When **3** and **4** were condensed with benzaldehyde their respective benzylidene derivatives **6** [51] and **7** were obtained (Scheme 2).

The spectral data of **6** and **7** show the disappearance of CH_2_ protons observed with the respective starting precursors **3** and **4** at δ~ 3.84 ppm, and the appearance of a δ-^1^H benzylidene C=CH at 8.62 ppm (^1^H-NMR) along with other δ-^1^H signals and ν_max_ absorption bands detected in the respective regions (Experimental section). Compounds **6** and **7** exhibited molecular ion peaks [M^+^] at *m/z* 380 and [M^+^−1] at *m/z* 426, respectively. 

Aiming to produce novel azo dyes and dye precursors with biological activities for dyeing and/or finishing of textile fibers, we considered the regioselectivity for attack on the key precursors **2**, **3** and **4** by different reagents. Thus, subjecting the title precursors to electrophilic or nucleophilic attack on their respective cyano-, ethoxycarbonyl-, and phenylcarbamoyl acetamido moieties, a variety of highly functionalized acyclic or heterocyclic molecules were obtained. When the intermediates **2**, **3** and **4** were subjected to coupling with diazotized aryl (heteraryl) amines, the corresponding cyano-, ethoxy-carbonyl-, or phenylcarbamoyl azo (hydrazone) dyes **8a-d**, **9a-e** and **10** were produced (Scheme 3). The dyes may exist in two possible tautomeric forms, namely, the azo-enol form (**A**) and the hydrazo-keto form (**B**). The dyes revealed common features indicating their existence in the azo-hydrazone form, whereby they exhibited intense amidic C=O absorptions around 1640 cm^−1^ (IR), δ-^1^H singlets about 12.80 ppm corresponding to a tautomeric hydrazone NH, along with singlets at δ ~ 10.88-15.70 ppm assigned to an enolic OH (^1^H-NMR). Other ν_max _values due to CN, ethoxycarbonyl C=O, phenyl-carbamoyl C=O (IR) in addition to δ-^1^H signals exhibited for four cyclohexene CH_2_s, amidic NH, ester CH_3_ and CH_2_ as well as phenyl aromatic protons (^1^H-NMR) were detected in the respective spectral regions. The mass spectra of the dyes revealed molecular ion peaks [M^+^] in agreement with their molecular formulae.

Treatment of the key precursors **2**, **3** and **4** with active methylene reagents (XCH_2_Y; X=Y=CN; X=CN, Y=CO_2_Et; X=Y=COCH_3_; X=COCH_3_, Y=CO_2_Et) afforded the respective 2-oxopyridine derivatives **11a-d**, **12a-b**, **13a-b** (Scheme 4). The reaction took place *via* 1,3-dinucleophilic attack by the active methylene reagent on the acetamido 1,3-bielectrophilic moiety of the starting materials. The synthesis of compounds **11a-d** and their analytical data were previously described by our group [42].

All data for compounds **12a-b**, **13a-b** were consistent with the proposed cyclization mechanism. The absence of the cited δ-^1^H CH_2_ singlets for the acetamido methylene protons observed with the respective precursors **3**, **4** at δ~ 3.84 ppm and the appearance of the pyridine C5-H protons at δ 7.92, 6.90, 6.92, 6.90 ppm in their respective ^1^H-NMR spectra confirmed the proposed structures. Moreover, δ-^1^H signals for OH were integrated at δ 11.76, 11.75 ppm (^1^H-NMR) for compounds **12b**, **13b**. Compounds **12a**, **12b** revealed δ-^1^H triplets about δ 1.13 ppm and δ-^1^H quartets about δ 3.56 ppm due to the ester CH_3_ and CH_2_ protons, respectively.

Additionally two carbonyl stretching modes due to a pyridine oxo function and an ethoxycarbonyl or phenylcarbamoyl C=O were observed for compounds **12a-b**, **13a-b,** in the 1673-1630 and 1663-1600 cm^−1^ regions, respectively. Mass spectral analysis displayed molecular ion peaks at *m/z* 357 [M^+^−1], 359 [M^+^] and 405 [M^+^] corresponding to the respective molecular formulae C_17_H_18_N_4_O_3_S for compound **12a**, C_17_H_17_N_3_O_4_S for **12b** and C_21_H_19_N_5_O_2_S for **13a**.

When 2-oxopyridine dye precursors **11a-d**, **12a-b** were coupled with diazotized aryl amines, the corresponding 2-oxo-5-pyridine azo (hydrazone) dyes **14a-d**, **15a-b** were obtained (Scheme 5). On the other hand, by subjecting the cyanoacetamido azo (hydrazone) dye **8a** to cyclization reaction *via* treatment with methylene carbonitrile reagents (XCH_2_CN; X=CN; X=CO_2_Et), the respective 2-oxo-3-pyridine azo (hydrazone) dyes **16a-b** were obtained (Scheme 5). The reaction followed 1,4-dipolar cyclization by **8a** with the methylene carbonitrile 1,2-dipoles.

Studying the absorption spectra of the resulting dyes, it is worthy of mention that, upon introduction of an aryl diazenyl group into the enaminone dye precursors **11a-d**, **12a-b**, the structural potential increases even more as the azo coupling products were found to exist in various tautomeric forms. The resulting dyes may exist as azo-keto; azo-enamino, hydrazone-keto, hydrazone imino, and azo-enol form species. Thus, the IR spectra of the dyes **14a-d**, **15a-b**, **16a-b** exhibited a common ring carbonyl absorption shifted to a low frequency domain around 1675 cm^−1^ due to conjugation of the C=O with the -C=C- in the enaminone system and/or the possibility of hydrogen bond formation (compounds **16a-b**, Scheme 11). Additionally, the azo-hydrazone tautomer is evident in the ^1^H-NMR spectra of the dyes, where the H peak of the hydrazone imine characteristically appearing at 12.35 ppm. The mass spectra of the dyes revealed [M^+^] ion peaks which corresponded to their expected molecular formulae.

Other 2-oxopyridine systems **17a-b**, were produced *via* treatment of the start precursor **3** with benzylidene carbonitrile reagents [51] (PhCH=C(CN)X; X=CN; X=CO_2_Et) (Scheme 6).

The reaction took place through β-attack followed by 1,6-dipolar intramolecular cyclization with concomitant aromatization. 

At the other extreme, the reaction of the start precursor **4** with phenyl isothiocyanate in basic dimethylformamide followed by heterocyclization using α-halo carbonyl reagents (XCH_2_-C(=O)R; X=Cl, R=CO_2_Et; X=Br, R=Ph; X=Cl, R=CH_3_) afforded the polyfunctional thiophene or thiazole derivatives **18a-b, 19**, respectively (Scheme 7). The reaction product depended on the nature of the α-halocarbonyl reagent [44]. The mechanism of reaction involved the intermediate formation of the potassium sulphide salt A. The disappearance of δ-^1^H acetamido CH_2_ singlet observed with the precursor **4** as revealed from the ^1^H-NMR spectra of **18a-b**, **19** and the appearance of ester CH_3_ triplets at δ 1.16 ppm and a CH_2_ quartet at δ 4.08 ppm for compound **18a**, along with the existence of a δ-^1^H singlet at 6.62 ppm assigned to the thiazole C5-H proton in compound **19** were sufficient proof for the proposed structures. Moreover the mass spectra of **18a-b** displayed molecular ion peaks [M^+^+1] at *m/z* 543 and [M^+^] at 574 confirming their molecular formulae C_29_H_26_N_4_O_3_S_2_, C_33_H_26_N_4_O_2_S_2_ and C_28_H_24_N_4_O_2_S_2_, respectively. 

At the other extreme, polyfunctional thiophene derivative **20** was designed *via* a reaction of the key precursor **3** with elemental sulfur and malononitrile (Gewald Pathway). The reaction took place through the intermediate formation of A and B. The latter suffered intramolecular 1,5-dipolar cyclization to afford the desired thiophene derivative **20** (Scheme 7). The ^1^H-NMR spectrum of **20** revealed the existence of a D_2_O exchangeable NH_2_ singlet at δ 3.82 ppm, as well as the appearance of δ-^1^H singlet at 11.79 ppm assigned to an OH proton. Moreover, in the mass spectrum of **20** the [M^+^] ion at *m/z* 344 confirmed the molecular formula C_15_H_12_N_4_O_2_S_2_.

On the other hand, when **3** was reacted with either hydrazine hydrate or phenyl hydrazine, the corresponding 3-oxopyrazole systems **21a-b** were produced (Scheme 8). The reaction involved 1,2-dinucleophilic cyclization by the hydrazine reagents on malonamic ester bielectrophilic moiety in **3**. Similarly when **9a** was subjected to the same reaction, the corresponding 5-oxopyrazole azo dyes **22a-b** were formed according to the same aforementioned mechanism (Scheme 8). Microanalysis and spectral data of the pyrazole systems **21a-b**, **22a-b** were fully consistent with the proposed structures. The existence of a common carbonyl absorption around 1672 cm^−1^ corresponding to the 3-oxopyrazole function (IR), the disappearance of the δ-^1^H ester CH_3_ and CH_2_ signals detected with the starting acyclic precursor **9a** (^1^H-NMR of **22a**) and the appearance of the-2-pyrazole phenyl multiplet (along with the phenyl hydrazone multiplet) at δ 6.22-7.65 ppm (^1^H NMR of **22b**) were considered as a definite proof for the proposed pyrazole systems. The mass spectra of the synthesized pyrazole systems displayed molecular ion peaks [M^+^+2] at *m/z* 262 for compound **21a**, [M^+^] at *m/z* 336 for **21b**, [M^+^+1] *m/z* 365 for **22a** and [M^+^+2] at *m/z* 442 for **22b**. The data corresponded to respective molecular formulae C_12_H_12_N_4_OS, C_18_H_16_N_4_OS, C_18_H_16_N_6_OS and C_24_H_20_N_6_OS.

In continuation of our aim for tailoring new functional heterocyclic targets with biological activity, we focused our study to a cyclization reaction of the acyclic hydrazone dye **10** with methylene carbonitrile reagents (XCH_2_CN; X=CN; X=CO_2_Et) in the presence of a catalytic amount of triethylamine. The reaction afforded the functionalized 1,3-oxazine azo dyes **23a-b**, respectively (Scheme 9).

The proposed mechanism involves nucleophilic attack by the enolic tautomer of **10** (azo-enol form) followed by 1,6-dipolar cyclization. The reaction took place through the non-isolable intermediate **A**. The data obtained from the IR, ^1^H-NMR, and MS spectra for the oxazine systems **23a-b** confirmed the proposed cyclization mechanism. Both dye systems displayed, in their respective ^1^H-NMR spectra oxazine C5-H protons, both at δ 6.92 ppm, as well as two D_2_O exchangeable NH_2_ singlets, both at δ 3.64. Compound **23a** exhibited a δ-^1^H singlet at 8.27 ppm due to the oxazine C4-imino function, while **23b** showed ν_max_ at 1662 cm^−1^ due to the oxazine C4-oxo absorption. The mass spectra of **23a-b** exhibited [M^+^] ion peaks at *m/z* 509 and [M^+^+1] at 511, respectively, indicating their corresponding molecular formulae C_27_H_23_N_7_O_2_S and C_27_H_22_N_6_O_3_S. 

Next, we moved to study the reaction of the acyclic hydrazone dyes **9a** and **10** with phenyl isothiocyanate in 1,4-dioxane containing a catalytic amount of triethylamine. The reaction involved a nucleophilic attack by the amidic NH function in **9a** and **10** on the C=S terminal of the isocyanate reagent to produce the acyclic intermediate A. the latter then underwent 1,6-dipolar cyclization through elimination of EtOH or H-OH to afford the functionalized pyrimidine azo (hydrazone) dye systems **24a** and **24b**, respectively (Scheme 10).

The analytical and spectral data of the dyes **24a-b** were in agreement with the proposed structures. Dye **24a** revealed two C=O absorptions at 1665, 1620 cm^−1^ corresponding to the 4,6-pyrimidinedioxo functions, while **24b** exhibited a 6-oxopyrimidine carbonyl absorption at 1664 cm^−1^. Both dyes showed, in their ^1^H-NMR spectra a singlet at δ~ 11.80 ppm due to the tautomeric hydrazone NH. Other ν_max_ absorptions and δ-^1^H values characterizing the proposed structures were integrated at the respective fields. The mass spectra of **24a-b** displayed [M^+^–1] ion peaks at *m/z* 484 and [M^+^+1] at *m/z* 561, indicating the molecular formulae C_25_H_19_N_5_O_2_S_2_ and C_31_H_24_N_6_OS_2_, respectively.

### 2.2. Spectral Characterization, Colour Assessment and Dyeing Properties

The dyes under investigation are based on 2-cyano-*N*-(3-cyano-4,5,6,7-tetrahydrobenzo[*b*]thiophen-2-yl)-acetamide, 2-ethoxycarbonyl-*N*-(3-cyano-4,5,6,7-tetrahydrobenzo[*b*]thiophen-2-yl)-acetamide or 2-phenylcarbamoyl-*N*-(3-cyano-4,5,6,7-tetrahydrobenzo[*b*]thiophen-2-yl)-acetamide. The dyes have their chromophoric system either attached to the acetamido terminus (acyclic dyes **8a-d**, **9a-e**, **10**, scheme 3), or directly linked to their respective cyclized precursors (heterocyclic dyes based on pyridine **14a-d**, **15a-b**, **16a-b**, Scheme 5; pyrazole **22a-b**, Scheme 8; oxazine **23a-b**, Scheme 9; and pyrimidine **24a-b**, Scheme 10). 

The present discussion will describe the effect of the structural configuration of these dyes on the shifts of ultraviolet-visible absorption maxima and the intensity of colour and fastness properties. Data of UV/vis absorption maxima, fastness and optical properties, as well as the colour shades on tested fabrics (nylon 66, acetate and polyester) are listed in Table 1.

**Table 1:** Fastness properties of disperse dyes on different fabrics.

Dye		K/S**	Fastness to Rubbing		Washing fastness		Fastness to Perspiration				Light	
	Sample	at λ_max_ =			at 90 ºC		Acidic		Alkaline			λ_max_
	Dyed *	400 nm	Dry	Wet	Alteration	Staining	Alteration	Staining	Alteration	Staining		nm
**8a**	N	24.01	3	4	3-4	3-4	5	4-5	5	4	3	741,
	A	4.78	4	4	5	5	5	5	5	5	2-3	392,
	P	6.61	4	4-5	5	5	5	5	5	5	2-3	223
**8b**	N	21.74	3	3-4	3	3-4	4-5	4-5	4-5	4-5	3-4	740,
	A	7.72	3	4	5	5	5	5	5	5	3	408,
	P	11.87	3	3-4	4-5	4-5	5	5	5	5	3	224
**8c**	N	24.13	3-4	4	3	3-4	5	4-5	5	4	3	741,
	A	9.29	4	5	5	5	5	5	5	5	2-3	404,
	P	13.81	3-4	4-5	5	5	5	5	5	5	2-3	225
**8d**	N	20.56	3-4	3	3	4	5	5	5	5	2-3	741,
	A	9.12	3	4	5	5	5	5	5	5	2-3	419,
	P	12.79	3	4	5	5	5	5	5	5	2-3	224
**9a**	N	10.33	3	3-4	5	5	5	5	5	5	4	740
	A	4.44	3-4	4	5	5	5	5	5	5	4-5	359
	P	7.02	3	3-4	5	5	5	5	5	5	4	237
**9b**	N	13.34	3	3-4	5	5	5	5	5	5	3-4	740
	A	4.59	3-4	4-5	5	5	5	5	5	5	4	364
	P	7.80	3-4	4	5	5	5	5	5	5	4	247
**9c**	N	8.73	3	3-4	5	5	5	5	5	5	4	647,
	A	3.91	4	4-5	5	5	5	5	5	5	3-4	364,
	P	9.08	3	3-4	5	5	5	5	5	5	3-4	242
**9d**	N	14.40	3	3-4	4-5	3	5	5	5	5	3	827
	A	4.82	3-4	4-5	5	5	5	5	5	5	3	323,
	P	8.75	3	3-4	5	5	5	5	5	5	3	229
**9e**	N	9.96	3-4	3	4-5	4-5	5	5	5	5	3	740,
	A	3.03	3	4	4-5	4-5	5	5	5	5	3	301,
	P	2.94	3	4	5	5	5	5	5	5	3	205
**10**	N	19.84	3	3-4	4-555	4-5	5	5	5	5	3	740,
	A	9.40	3-4	4-5		5	5	5	5	5	3	390,
	P	15.91	3	4		5	5	5	5	5	3-4	238
**14a**	N	22.93	5	4-5	3-4	3-4	5	4-5	5	4-5	3	741,
	A	8.87	5	5	5	5	5	5	5	5	3	405,
	P	15.52	5	5	5	5	5	5	5	5	3-4	224
**14b**	N	24.01	4-5	5	3	4	5	4-5	5	4-5	3	740,
	A	9.66	5	5	5	5	5	5	5	5	3	405,
	P	16.08	5	5	5	5	5	5	5	5	3-4	232
**14c**	N	11.46	3	3-4	4-5	4-5	5	5	5	5	2-3	740
	A	5.77	3	4	5	5	5	5	5	5	2-3	401
	P	7.00	3	4	5	5	5	5	5	5	2-3	
**14d**	N	24.78	4-5	4	4	3	5	4-5	5	4	3	740,
	A	8.98	3-4	4-5	5	5	5	5	5	4-5	3-4	401,
	P	12.53	3-4	4	5	5	5	5	5	5	3-4	222
**15a**	N	17.19	3-4	4	4-5	4-5	5	5	5	5	3	740,
	A	6.08	3-4	4	5	5	5	5	5	5	3-4	219
	P	11.07	3	4	5	5	5	5	5	5	3	
**15b**	N	14.94	3	3-4	4-5	4-5	5	5	5	5	3	740,
	A	6.51	3	4	5	5	5	5	5	5	3	224
	P	9.94	3	4	5	5	5	5	5	5	3	
**16a**	N	25.19	3	3-4	4	4	5	4-5	5	4-5	3	741,
	A	9.04	3	4	5	5	5	5	5	5	3	395,
	P	14.31	3-4	4	5	5	5	5	5	5	3	221
**16b**	N	23.28	3-4	4	3-4	3-4	4-5	4-5	4-5	4-5	3	741,
	A	7.83	3	4	5	5	5	5	5	4-5	3-4	400,
	P	11.40	3	4	5	5	5	5	5	5	3	223
**22a**	N	16.62	3	3-4	4-5	4-5	5	5	5	5	3-4	741,
	A	7.06	3	3-4	5	5	5	5	5	5	4-5	370,
	P	11.07	3	3	5	5	5	5	5	5	4-5	224
**22b**	N	18.32	3	4	5	5	5	5	5	5	3-4	741,
	A	8.32	3	4	5	5	5	5	5	5	4-5	364,
	P	13.02	3	3-4	5	5	5	5	5	5	4-5	224
**23a**	N	15.52	4	5	4-5	4-5	5	5	5	5	3	741,
	A	6.87	4	5	5	5	5	5	5	5	3	235
	P	9.71	4	4-5	5	5	5	5	5	5	3-4	
**23b**	N	19.93	3	3-4	4-5	4-5	5	5	5	5	3	740,
	A	8.94	3	4	5	5	5	5	5	5	3-4	235
	P	14.45	3	3-4	5	5	5	5	5	5	4	
**24a**	N	5.56	3	4	5	5	5	5	5	5	3-4	741,
	A	4.22	3-4	4	5	5	5	5	5	5	4-5	230
	P	4.43	3	4	5	5	5	5	5	5	4-5	
**24b**	N	18.24	3-4	3-4	5	5	5	5	5	5	4	740,
	A	8.22	3	4	5	5	5	5	5	5	3-4	223
	P	12.98	3	4	5	5	5	5	5	5	4	

* N, Nylon 66; A, Acetate; P, Polyester. ** K/S = (1-R)^2^/2R. R: a decimal fraction of reflection of the dyed fabric. K: absorption coefficient. S: scattering coefficient.

#### 2.2.1. Spectral Characterization

The target dyes revealed analytical and spectral data in accordance to their molecular structures. Most dyes exist in the azo-hydrazone tautomeric structure, as revealed by spectral data. The possibility of H bond formation between the imine-H of the hydrazone tautomer and the carbonyl bond (Scheme 11) as well as presence of a C=O moiety in an enaminone conjugate system would shift the carbonyl absorption band to a low frequency domain (ν_max_ 1610-1600 cm^−1^). Additionally, the azo-hydrazone tautomer is evident in the ^1^H-NMR spectra where the H peak of the imine group characteristically appears at δ ~12.00 ppm. The UV/vis absorption maxima of the synthesized dyes were tested in the aqueous solution [after adding dimethyl sulphoxide (1.00/20.00 mL) to improve the water solubility]. From the analysis of the UV/vis absorption data, it was found that, for each inter-related system, none of the dyes changed significantly with respect to each other. 

The bathochromic shift observed for λ_max_ values of some dyes could be attributed to more extensive delocalization within each system configuration which in turn, is dependent on the presence of electron donating substituents. Suitable electronic absorptions were found in the blue violet region of 390-420 nm. An absorption band below 250 nm was detected for most dye systems which could be relatable to electron transition through a possible azo ligand resulting from intramolecular chelation by H-bond (Scheme 11).

A common absorption band appearing around 740 nm was observed in the UV/vis spectra of nearly all synthesized dyes which could arise from a transition involving electron migration along the entire conjugate enaminone and/or enaminonitrile configuration. The UV/vis absorption data showed no significant bathochromic or hypsochromic shifts when measured in ethanol as a solvent. 

#### 2.2.2. Colour Assessment and Dyeing Properties

The novel dyes were applied to nylon 66, acetate and polyester fabrics at 5% depth by the high-temperature-pressure technique and gave generally bright, intense golden yellow, mustard yellow, orange, dark orange, brick red, brick brown, pale green and bright green hues. Due to the good migration properties of these disperse dyes, leveling agents were not required. The dyed fabrics were analyzed on qualitative tests by estimation fastness shades with grey scale, the results were expressed in terms of colour ratings 1-5 (Table 1).

In general, the data revealed that wash fastness rating for change in colour as well as staining of adjacent fabrics are very good (4-5). Rubbing fastness of the samples, assessed in terms of dry and wet rubbing indicated good fastness to rubbing for both dry and wet (3-5). Perspiration fastness properties (acidic and alkaline) of the dyed samples in terms of ratings for staining of adjacent fabrics and change are very good (4-5). The high ratings for change in colour at both acidic and alkaline conditions indicate that the sensitivity of the dyed samples are not related to PH. This may be due to the stability of the dyes towards degradation under either acidic or basic conditions. 

Light fastness of the dyeing was of a generally of good order (3-5). The lightness ratings were primarily influenced by the depth of the hue. The data for colour yield expressed as K/S indicated higher values on nylon in comparison to other fabrics. This is probably due to the high substantivity of the dyes on nylon. 

From the aforementioned data, it is noteworthy that the fastness properties for the dyes under investigation are inter-related since they depend, among other factors, on the rate of diffusion of the dye in the fabric. This rate is a function of the geometry of the dye molecule. Fastness to rubbing depends on the presence of loose dye particles on the fabric surface. The washing and perspiration fastness is, to a certain extent, dependent on the substantivity of the dye for the fabric which determines its tendency to partition in favour of the fabric.

### 2.3. Biology

#### 2.3.1. Antimicrobial Evaluation of the Newly Synthesized Compounds

The newly synthesized dyes and dye precursor were screened *in vitro* for their antibacterial and antifungal activities against selected microbial strains, namely *Escherichia coli ECT 101* (Gram-negative bacteria), *Bacillus subtilis CECT 498* and *Bacillus cereus CECT 148* (Gram-positive bacteria) and *Candida albicans 1394* (a representative fungal species).

The minimal inhibitory concentration (MIC) in μg/mL was determined using an adapted agar streak dilution method based on radial diffusion [45,46]. Under the same conditions, solutions of control antibiotics ampicillin (antibacterial) and cycloheximide (antifungal) were used as standards. 

The antimicrobial screening results of the synthesized compounds and standard antibiotics are given in Table 2. Structure-antimicrobial (biological) activity relationship for the newly synthesized compounds were studied and determined against the selected test bacterial and fungal strains. 

**Table 2:** Antimicrobial and antifungal evaluations of the synthesized compounds MIC (mg/mL).

Compound	*E. coli*	*B. cereus*	*B. subtilis*	*C. albicans*
**3**	NA	4.85	12.00	10.00
**4**	18.51	22.16	20.18	10.00
**5**	6.60	17.81	6.80	8.00
**6**	NA	14.62	4.18	0.60
**7**	6.58	8.48	4.46	12.38
**8a**	NA	4.22	10.89	12.42
**8b**	NA	4.39	4.33	12.77
**8c**	NA	0.08	2.22	6.44
**8d**	NA	3.03	0.68	20.50
**9a**	NA	16.32	6.22	0.40
**9b**	NA	12.30	4.22	12.55
**9c**	NA	6.03	12.42	4.55
**9d**	NA	12.34	6.13	0.40
**9e**	NA	2.25	14.00	16.00
**10**	NA	7.39	4.33	12.77
**12a**	16.77	6.28	22.48	4.66
**12b**	4.55	10.33	2.26	10.58
**13a**	12.57	8.00	4.62	33.00
**13b**	NA	4.76	8.66	8.22
**14a**	16.60	12.42	4.01	0.20
**14b**	0.36	1.16	9.67	0.04
**14c**	10.50	4.77	8.80	30.55
**14d**	4.72	19.22	6.80	8.06
**15a**	10.21	2.36	0.22	0.20
**15b**	NA	12.30	4.22	12.55
**16a**	NA	1.25	8.23	2.24
**16b**	NA	0.08	2.22	6.44
**17a**	NA	2.19	6.44	4.48
**17b**	10.55	6.88	5.38	8.79
**18a**	6.44	8.46	6.42	0.61
**18b**	8.99	12.68	6.77	2.87
**19**	6.48	8.18	22.30	18.42
**20**	10.33	4.14	10.33	6.08
**21a**	NA	12.34	6.13	0.40
**21b**	8.22	6.03	12.42	4.55
**22a**	8.48	6.08	18.30	16.48
**22b**	NA	6.18	4.03	0.81
**23a**	NA	6.88	5.38	6.99
**23b**	6.98	6.68	4.36	8.26
**24a**	19.04	8.36	10.60	18.26
**24b**	12.41	8.52	2.01	0.80
**Ampicillin**	6.25	3.13	12.50	–
**Cycloheximide**	–	–	–	12.50

NA: not active.

According to this study it was observed that the MIC values of the dyes and dye precursors are generally within the 0.04-30.00 μg/mL range against all tested microbial organisms, which reveals significant and potent antimicrobial activity (Table 2). However, a number of the synthesized compounds (dyes **8a-d**, **9a-e**, **15b**, **16a-b**, **22b**, **23a** and dye precursors **3**, **13b**, **17a**, **21a**) were found to be totally inactive towards the Gram-negative bacterial species *E. coli.* This could be related to the presence of electron withdrawing functions (CN, CO_2_Et, CONHPh, N=N-) conjugated with electron donating groups (NH_2_, OH, =N-NH). The increase in electron attracting character of these compounds cause a relative decrease in their biological activity. Furthermore, the intricate nature of the cell wall of this Gram-negative bacterial strain compared with Gram-positive test bacteria *B. subtilis* and *B.cereus* may inhibit the adsorption of most compounds onto the surface and consequently revealed weaker susceptibility for these compounds.

On the other hand, as an impressive result, the active concentration of almost all synthesized dyes and dye precursors against tested Gram-positive bacteria was found to be similar or even lower values compared with control antibiotics against these strains (Table 2). This may be attributed to the presence of an oxo function in the main structure of the dyes and dye precursors which could be responsible to bind or complex with nucleophilic amino acids in proteins leading to inactivation of the microorganisms and loss of function. Additionally, the presence of an oxo function in the synthesized compounds may lead to the formation of H-bonds with water molecules which enable these compounds to more readily form positive ions in aqueous solution thereby inhibiting microbial growth by adsorption onto the bacterial surface [47]. Also, it is worth of mentioning that a high level of antifungal activities was observed for all tested compounds against the selected yeast *C. albicans* (Table 2). The dyes **8a**, **8c**, **9a**, **9a-d**, **10**, **14a-b**, **14d**, **15a**, **16a-b**, **22b**, **23a-b**, **24b** and dye precursors **3**, **4**, **5**, **6**, **7**, **12a-b**, **13a-b**, **17a-b**, **18a-b**, **20**, **21a-b** all exhibited superior antifungal activity than cycloheximide against this fungal strain.

#### 2.3.2. Antimicrobial Evaluation of the Dyed Fabrics

Selected dyed fabrics (nylon 66 dyed with **8a**, **9d**, **9e**, **14c**, **14d**, **16a**, **22a**, **24b**, acetate dyed with **8b**, **8d**, **9a**, **14a**, **15b**, **16b**, **22b**, **23b** and polyester dyed with **8c**, **9b**, **9c**, **10**, **14b**, **15a**, **23a**, **24a**) were chosen to study their biological activities towards *E. coli* and *Pseudomonas aeruginosa* (Gram-negative bacteria), *B. subtilis,* *Staphylococcus aureus* and *B. cereus* (Gram-positive bacteria) and *C. albicans* (a representative fungal species). Diameters of the inhibition zones in mm were measured. The data of the disc susceptibility tests showed significant and potent antimicrobial activity of the dyed fabrics against all tested organisms (Table 3).

**Table 3:** Antimicrobial and antifungal potentialities of the tested dyed fabrics express as size (mm) of inhibition zones.

Dye	Sample Dyed *	*E. coli*	*B. cereus*	*B. subtilis*	*C. albicans*	*P. aeruginosa*	*S. aureus*
**8a**	N	20	18	17	27	17	15
**8b**	A	12	16	18	22	12	16
**8c**	P	15	12	16	23	19	18
**8d**	A	12	10	13	22	14	14
**9a**	A	15	11	15	22	18	15
**9b**	P	10	12	11	20	12	16
**9c**	P	18	11	10	28	14	18
**9d**	N	20	14	18	22	18	12
**9e**	N	18	20	18	28	20	15
**10**	P	16	15	16	25	16	20
**14a**	A	16	11	10	18	14	15
**14b**	P	14	13	15	19	14	17
**14c**	N	14	12	14	19	14	17
**14d**	N	10	10	17	28	14	14
**15a**	P	14	12	14	19	13	16
**15b**	A	18	14	18	16	20	22
**16a**	N	19	19	19	25	20	20
**16b**	A	20	20	18	20	19	19
**22a**	N	15	18	16	24	14	18
**22b**	A	15	14	15	24	16	19
**23a**	P	15	13	15	24	15	15
**23b**	A	18	18	18	20	19	15
**24a**	P	19	19	19	30	20	20
**24b**	N	14	14	12	20	16	16

* N, Nylon 66; A, acetate; P, polyester.

## 3. Experimental

### 3.1. General

#### 3.1.1. Equipment

All melting points were determined on an Electrothermal digital melting point apparatus and are uncorrected. IR spectra (KBr discs) were recorded on a FTIR plus 460 or Pye Unicam SP-1000 spectrophotometer. ^1^H-NMR spectra were recorded with Varian Gemini-200 (200 MHz) and Jeol AS 500 MHz instruments, ^13^C-NMR spectra were measured with the Jeol AS 500 MHz instrument. Both spectra were performed in DMSO-*d_6_* as solvent using TMS as internal standard and chemical shifts are expressed as δ ppm. MS (EI) spectra were recorded with Hewlett Packard 5988 A GC/MS system and GCMS-QP 1000 Ex Shimadzu instruments. UV/vis absorption maxima (λ_max_) were recorded on UV-16600 Series. Analytical data were obtained from the Micro-analytical Data Unit at Cairo University and were performed on Vario EL III Elemental analyzer. The dyeing operation was carried out using Wemer Mathis AG Textimaschine-Laborapparate CH-8155 Niederhasli/Zürich apparatus. Colour strength (K/S) of the dyed samples was measured by using OPTIMATCH 3100. The colour fastness to washing was determined using Launder-ometer. Colour fastness to rubbing was determined using Crock-Meter Type FD II and colour fastness to perspiration was determined using Prespiration Tester. The light fastness test was measured by using Mercury-Tungsten lamp. 

#### 3.1.2. Materials and Chemicals

Pretreated fabrics used throughout this work; namely cellulose triacetate, nylon 66 and polyester (polyethylene glycol terephthalate) were supplied by Misr-Helwan Co. for spinning and weaving, Helwan, Cairo, Egypt. Dispersing agents namely Remol-HT was supplied by Hoechst (Germany). *2-Cyano-N-(3-cyano-4,5,6,7-tetrahydrobenzo[b]thiophen-2-yl)-acetamide* (**2**) was synthesized following the method previously described by our group [42]. *N-(3-cyano-4,5,6,7-tetrahydrobenzo[b]thiophen-2-yl)-malonamic acid ethyl ester* (**3**) was synthesized according to a method described [50].

### 3.2. Chemistry

*N-(3-Cyano-4,5,6,7-tetrahydrobenzo[b]thiophen-2-yl)-N^\^-phenyl-malonamide* **(4**): Equimolar amounts of **3** (2.92 g, 0.01 mol) and aniline (0.93 g, 0.01 mol) in dimethylformamide (20 mL) were heated under reflux for 5 h. The solid product formed upon pouring onto ice/water mixture was collected by filtration and crystallized from dimethylformamide. Yellowish white crystals, m.p. 147-150 °C, yield: 2.95 g (87%); Anal. For C_18_H_17_N_3_O_2_S (339.41), (% Calcd./Found): 63.70/64.01 (C), 5.05/5.33 (H), 12.38/12.60 (N), 9.45/9.70 (S); IR (*ν*, cm^−1^): 3430-3142 (2NH), 3083 (CH aromatic), 2937-2839 (CH_2_), 2198 (CN), 1696, 1663 (2C=O), 1524,1440 (C=C); ^1^H-NMR (δ, ppm): 1.72-2.91 (m, 8H, cyclohexene 4CH_2_), 3.89 (s, 2H, CH_2_), 6.95-7.65 (m, 5H, C_6_H_5_), 11.54, 11.81 (2s, 1H each, 2NH); ^13^C NMR (δ, ppm): 23.8, 24.0, 28.2, 29.3 (4 CH_2_), 40.5 (CH_2_), 93.1 (thiophene-C3), 114.7 (CN), 117.3, 118.8, 119.6, 120.3, 129.7 (C_6_H_5_), 131.2, 131.6, 146.8 (thiophene 3C), 163.2, 165.6 (2 C=O); MS *m/z* (%): 341 [M^+^+2] (8.00), 340 [M^+^+1] (12.50), 339 [M^+^] (49.70), 178 (100.00), 93 (74.90), 77 (56.20).

*2-(1H-Benzoimidazol-2-yl)-N-(3-cyano-4,5,6,7-tetrahydrobenzo[b]thiophen-2-yl)-acetamide* (**5**): Equimolar amounts of **3** (2.92 g, 0.01 mol) and *o*-phenylenediamine (1.08 g, 0.01 mol) were heated in an oil bath at 140 °C for 1h. The reaction mixture was then boiled in ethanol (30 mL) for few minutes, poured onto ice/water mixture and the formed product was crystallized from 1,4-dioxane. Pale brown crystals, m.p. 94-98 °C, yield: 2.35 g (70%); Anal. For C_18_H_16_N_4_OS (336.41), (% Calcd./Found): 64.26/64.51 (C), 4.79/4.81 (H), 16.65/16.30 (N), 9.53/9.82 (S); IR (*ν*, cm^−1^): 3392 (2NH), 3050 (CH aromatic), 2924-2844 (CH_2_), 2196 (CN), 1607 (C=O), 1544, (C=N), 1502, 1431(C=C); ^1^H-NMR (δ, ppm): 1.77-2.93 (m, 8H, cyclohexene 4CH_2_), 4.44 (s, 2H, CH_2_), 6.88-7.63 (m, 4H, C_6_H_4_), 8.37, 10.54 (2s, 1H each, 2NH); ^13^C-NMR(δ, ppm): 23.3, 24.4, 28.4, 29.6 (4 CH_2_), 40.7 (CH_2_), 93.3 (thiophene-C3), 115.9 (CN), 121.0, 121.4, 122.2, 122.5, 127.4 (C_6_H_4_), 131.0, 131.8, 146.4 (thiophene 3C), 145.8 (imidazole-C2), 162.5 (C=O), 175.7 (C=N); MS *m/z* (%): 337 [M^+^+1] (55.42), 336 [M^+^] (100.00), 335 [M^+^–1] (41.43).

#### 3.2.1. Reaction of 4,5,6,7-Tetrahydrobenzo[*b*] Thiophene Derivatives **3** and **4** with Benzaldehyde: Synthesis of **6** and **7**

To a mixture of equimolar amounts of **3** (2.92 g, 0.01 mol) or **4** (3.39 g, 0.01 mol) in 1,4-dioxane (25 mL) containing piperidine (0.50 mL), benzaldehyde (1.06 g, 0.01 mol) was added. The reaction mixture was heated under reflux for 5h. The solid products formed upon pouring onto ice/water mixture were containing few drops of hydrochloric acid collected by filtration and crystallized from 1,4-dioxane.

*2-(3-Cyano-4,5,6,7-tetrahydrobenzo[b]thiophen-2-yl-carbamoyl)-3-phenyl-acryclic acid ethyl ester* (**6**): The data for compound **6 **has been published earlier by our group [51].

*2-Benzylidene-N-(3-cyano-4,5,6,7-tetrahydrobenzo[b]thiophen-2-yl)-N^\^-phenyl-malonamide* (**7**): Dark brown crystals, m.p. 84-88 °C, yield: 3.42 g (80%); Anal. For C_25_H_21_N_3_O_2_S (427.52), (% Calcd./Found): 70.24/69.90 (C), 4.95/5.20 (H), 9.83/10.12 (N), 7.50/7.82 (S); IR (*ν*, cm^−1^): 3282 (2NH), 3066 (CH aromatic), 2928-2851 (CH aliphatic), 2210 (CN), 1666, 1598, (2C=O) 1540,1441 (C=C); ^1^H-NMR (δ, ppm): 1.71-2.69 (m, 8H, cyclohexene 4CH_2_), 7.28-7.95 (m, 10H, 2C_6_H_5_), 8.62 (s, 1H, benzylidene CH), 10.14, 11.77 (2s, 1H each, 2NH); MS *m/z* (%): 427 [M^+^], 426 [M^+^–1] (36.83), 140 (74.67), 73 (100.00), 77 (1.52).

#### 3.2.2. Reaction of 4,5,6,7-Tetrahydrobenzo[*b*]thiophene Derivative **2** with Aryl Diazonium Chloride Salts: Synthesis of **8a-d**

To a cold solution (0-5 °C) of **2 **(2.45 g, 0.01 mol), in ethanol (20 mL) containing sodium hydroxide (1.00 g) an equimolar amount of diazotized aniline, diazotized *p*-chloroaniline, diazotized *p*-toluidene and diazotized *p*-methoxyaniline were gradually added while stirring. The solid products formed upon cooling in an ice-bath were collected by filtration, washed with water and crystallized from 1,4-dioxane.

*2-Cyano-2-(2-phenylhydrazono)-N-(3-cyano-4,5,6,7-tetrahydrobenzo[b]thiophen-2-yl)-acetamide* **(8a**): the data of compound **8a** has been published earlier by our group [42].

*2-[(4-Chlorophenyl)-hydrazono]-2-cyano-N-(3-cyano-4,5,6,7-tetrahydrobenzo[b]thiophen-2-yl)-acetamide* (**8b**): Reddish brown crystals, m.p. 133-138 °C, yield: 3.45 g (90%); Anal. For C_18_H_14_N_5_OSCl (383.85), (% Calcd./Found): 56.32/56.54 (C), 3.68/3.90 (H), 18.24/18.00 (N), 8.35/8.60 (S); IR (*ν*, cm^−1^): 3332-3218 (resonating OH, 2NH), 3090 (CH aromatic), 2932-2855 (CH_2 _cyclohexene), 2256, 2214 (2CN), 1627 (C=O), 1575, 1490 (C=C), 1535 (=N-NH); ^1^H-NMR (δ, ppm): 1.74-2.89 (m, 8H, cyclohexene 4CH_2_), 6.92-7.83 (m, 4H, C_6_H_4_), 11.80, 12.80 (2s, 1H each, 2NH), 15.70 (s, 1H, resonating OH) ; MS *m/z* (%): 385 [M^+^+2] (18.90), 383 [M^+^], 220 (13.50), 178 (16.20), 127 (64.90), 65 (100.00).

*2-Cyano-N-(3-cyano-4,5,6,7-tetrahydrobenzo[b]thiophen-2-yl)-2-(p-tolyl-hydrazono)-acetamide* (**8c**): Dark reddish brown crystals, m.p. 116-120 °C, yield: 2.00 g (55%); Anal. For C_19_H_17_N_5_OS (363.44), (% Calcd./Found): 62.79/63.16 (C), 4.71/4.99 (H), 19.27/18.90 (N), 8.82/9.04 (S); IR (*ν*, cm^−1^): 3330-3218 (resonating OH, 2NH), 3090 (CH aromatic), 2929-2857 (CH_3_,CH_2_ cyclohexene), 2253, 2218 (2CN), 1622 (C=O), 1574, 1443 (C=C), 1518 (=N-NH); ^1^H-NMR (δ, ppm): 1.17 (s, 3H, CH_3_), 1.71-2.84 (m, 8H, cyclohexene 4CH_2_), 6.88 (s, 1H, NH) 7.13-7.65 (m, 4H, C_6_H_4_), 11.99, (s, 1H, NH), 15.59 (s, 1H, resonating OH) ; MS *m/z* (%): 363 [M^+^], 150 (71.40), 77 (54.80), 50 (100.00). 

*2-Cyano-N-(3-cyano-4,5,6,7-tetrahydrobenzo[b]thiophen-2-yl)-2-[(4-methoxyphenyl)-hydrazono]acetamide* (**8d**): Dark reddish brown crystals, m.p. 90-94 °C, yield: 2.31 g (61%); Anal. For C_19_H_17_N_5_O_2_S (379.44), (% Calcd./Found): 60.14/59.80 (C), 4.52/4.82 (H), 18.46/18.10 (N), 8.45/8.82 (S); IR (*ν*, cm^−1^): 3325-3215 (2NH), 3090 (CH aromatic), 2932-2839 (CH_3_, CH_2_ cyclohexene), 2254, 2211 (2CN), 1610 (C=O), 1576, 1459 (C=C), 1511 (=N-NH); ^1^H-NMR (δ, ppm): 1.20 (s, 3H, CH_3_), 1.75-2.50 (m, 8H, cyclohexene 4CH_2_), 7.07-7.79 (m, 4H, C_6_H_4_), 10.80, 11.90 (2s, 1H each, 2NH), 12.43 (s, 1H, resonating OH); MS *m/z* (%): 379 [M^+^] (54.49), 300 (62.55), 61 (100.00).

#### 3.2.3. Synthesis of *N*-(3-cyano-4,5,6,7-tetrahydrobenzo[*b*]thiophen-2-yl)-malonamic Acid Ethyl Ester Azo Derivatives **9a-d**

To a cold solution (0-5 °C) of **3** (2.92 g, 0.01 mol), in ethanol (20 mL) containing sodium hydroxide (1.00 g) an equimolar amount of diazotized aniline, diazotized *p*-chloroaniline, diazotized *p*-toluidene or diazotized p-methoxyaniline was gradually added while stirring. The solid products formed upon cooling in an ice-bath were collected by filtration, washed with water and crystallized from 1,4-dioxane. 

*N-(3-Cyano-4,5,6,7-tetrahydrobenzo[b]thiophen-2-yl)-2-(phenylhydrazono)-malonamic acid ethyl ester* (**9a**): the data of compound **9a **has been published earlier by our group [51].

*2-[(4-Chlorophenyl)-hydrazono]-N-(3-cyano-4,5,6,7-tetrahydrobenzo[b]thiophen-2-yl)malonamic acid ethyl ester* (**9b**): Orange crystals, m.p. 186-190 °C, yield: 4.09 g (95%); Anal. For C_20_H_19_ClN_4_O_3_S (430.91), (% Calcd./Found): 55.75/55.42 (C), 4.44/4.11 (H), 13.00/13.34 (N), 7.44/7.73 (S); IR (*ν*, cm^−1^): 3439-3348 (2NH), 3159 (CH aromatic), 2932-2850 (CH_3_, CH_2_), 2208 (CN), 1664, 1627 (2C=O), 1590, 1453 (C=C), 1529 (=N-NH); ^1^H-NMR (δ, ppm): 1.81 (t, *J* = 6.90 Hz, 3H, ester CH_3_), 2.50-2.66 (m, 8H, cyclohexene 4CH_2_), 3.80 (q, *J* = 6.90 Hz, 2H, ester CH_2_), 7.42-7.74 (m, 4H, C_6_H_4_), 10.60, 12.50 (2s, 1H each, 2NH), 13.60 (s, 1H, resonating OH). 

*N-(3-Cyano-4,5,6,7-tetrahydrobenzo[b]thiophen-2-yl)-2-(p-tolyl-hydrazono)-malonamic acid ethyl ester* (**9c**): Pale orange crystals, m.p. 256-260 °C, yield: 3.94 g (96%); Anal. For C_21_H_22_N_4_O_3_S (410.49), (% Calcd./Found): 61.45/61.17 (C), 5.40/5.60 (H), 13.65/14.00 (N), 7.81/8.02 (S); IR (*ν*, cm^−1^): 3470-3163 (resonating OH, 2NH), 3090 (CH aromatic), 2932-2852 (CH_3_, CH_2_), 2210 (CN), 1665, 1630 (2C=O), 1570, 1454 (C=C), 1525 (=N-NH); ^1^H-NMR (δ, ppm): 1.20 (t, *J* = 7.70 Hz, 3H, ester CH_3_), 1.75 (s, 3H, CH_3_), 1.88-2.85 (m, 8H, cyclohexene 4CH_2_), 3.30 (q, *J* = 7.70 Hz, 2H, ester CH_2_), 7.20-7.62 (m, 4H, C_6_H_4_), 10.70, 12.59 (2s, 1H each, 2NH), 13.66 (s, 1H, resonating OH); MS *m/z* (%): 410 [M^+^] (18.90), 233 (29.70), 204 (13.50), 178 (37.80), 106 (100.00), 77 (70.30).

*N-(3-Cyano-4,5,6,7-tetrahydrobenzo[b]thiophen-2-yl)-2-[(4-methoxy-phenyl)-hydrazono]-malonamic acid ethyl ester* (**9d**): Deep orange crystals, m.p. 240-245 °C, yield: 3.54 g (83%); Anal. For C_21_H_22_N_4_O_4_S (426.49), (% Calcd./Found): 59.14/58.90 (C), 5.20/5.43 (H), 13.14/13.30 (N), 7.52/7.82 (S); IR (*ν*, cm^−1^): 3457-3188 (resonating OH, 2NH), 3078-3001 (CH aromatic), 2933-2841 (CH_3_, CH_2_), 2210 (CN), 1663, 1600 (2C=O), 1571, 1457 (C=C), 1530 (=N-NH) ; ^1^H-NMR (δ, ppm): 1.01 (t, *J* = 6.85 Hz, 3H, ester CH_3_), 1.71 (s, 3H, CH_3_), 1.87-2.85 (m, 8H, cyclohexene 4CH_2_), 3.70 (q, *J* = 6.85 Hz, 2H, ester CH_2_), 6.91-7.92 (m, 4H, C_6_H_4_), 11.78, 13.45 (2s, 1H each, 2NH), 14.93 (s, 1H, resonating OH); MS *m/z* (%): 426 [M^+^] (1.90), 249 (4.50), 204 (11.60), 178 (63.00), 150 (100.00).

*N-(3-Cyano-4,5,6,7-tetrahydrobenzo[b]thiophen-2-yl)-2-(3-cyano-4,5,6,7-tetrahydrobenzo[b]thiophen-2-ylazo)malonamic acid ethyl ester* (**9e**): Compound **9e** was synthesized according to the following method: A diazotized solution of 2-amino-3-cyano-4,5,6,7-tetrahydrobenzo[b]-thiophene (**1**) was prepared by dissolving **1** (1.78 g, 0.01 mol) in a mixture of acetic acid (10 mL) and propionic acid (2 mL) and stirring while cooling (5 °C) for 20 min. Concentrated sulphuric acid (5 mL) was then added, followed by portion-wise addition of sodium nitrite (0.69 g, 0.01 mol). The diazotized solution was heated (60-65 °C) while stirring for 1 h until a clear solution was obtained, cooled to 5 °C, and then added to a solution of **3** (2.92 g, 0.01 mol) in ethanol (35 mL) containing sodium hydroxide (1.00 g) while cooling in an ice bath. The reaction mixture was neutralized by pouring onto ice/water mixture containing few drops of hydrochloric acid. The solid product, thus formed, was collected by filtration and crystallized from ethanol. Brown crystals, m.p. 271-273 °C, yield: 3.85 g (80%); Anal. For C_23_H_23_N_5_O_3_S_2_ (481.59), (% Calcd./Found): 57.36/57.01 (C), 4.81/5.02 (H), 14.54/14.32 (N), 13.32/13.60 (S); IR (ν, cm^−1^): 3450-3193 (resonating OH, 2NH), 3085-3005 (CH aromatic), 2936-2859 (CH_3_, CH_2_), 2211, 2195 (2CN), 1678, 1628 (2C=O), 1573, 1464 (C=C), 1545 (=N-NH); ^1^H-NMR (δ, ppm): 1.20 (t, 3H, ester CH_3_), 1.75-2.58 (m, 16H, cyclohexene 8CH_2_), 3.85 (q, 2H, ester CH_2_), 6.90, 11.50 (2s, 1H each, 2NH), 11.77 (s, 1H, resonating OH); MS *m/z* (%): 481 [M^+^] (0.02), 163 (27.63), 135 (100.00), 105 (31.48), 77 (17.45).

*N-(3-Cyano-4,5,6,7-tetrahydrobenzo[b]thiophen-2-yl)-N^\^-phenyl-2-(phenylhydrazono)malonamide* (**10**): *Method (A)*: To a cold solution (0-5 °C) of **4** (3.39 g, 0.01 mol), in ethanol (20 mL) containing sodium hydroxide (1.00 g) an equimolar amount of diazotized aniline was gradually added while stirring. The solid product formed upon cooling in an ice-bath was collected by filtration, washed with water and crystallized from 1,4-dioxane. *Method (B)*: To a solution of **9a** (3.96 g, 0.01 mol) in dimethylformamide (30 mL), aniline (0.93 g, 0.01 mol) was added. The reaction mixture was heated under reflux for 5 h. The solid product formed upon pouring onto ice/water mixture was collected by filtration, washed with water and crystallized from 1,4-dioxane. Pale orange crystals, m.p. 106-110 °C, yield: 3.41 g (77% in method **A** and **B**); Anal. For C_24_H_21_N_5_O_2_S (443.52), (% Calcd./Found): 64.99/64.62 (C), 4.77/4.36 (H), 15.79/15.57 (N), 7.23/7.67 (S); IR (*ν*, cm^−1^): 3290-3209 (resonating OH, 3NH), 3063 (CH aromatic), 2934-2857 (CH_2_ cyclohexene), 2210 (CN), 1667, 1598, (2C=O) 1527,1443 (C=C); ^1^H-NMR (δ, ppm): 1.71-2.62 (m, 8H, cyclohexene 4CH_2_), 7.26-7.57 (m, 10H, 2C_6_H_5_), 10.14, 11.76, 13.35 (3s, 1H each, 3NH), 13.78 (s, 1H, resonating OH); MS *m/z* (%): 443 [M^+^] (3.50), 339 (18.10), 178 (100.00), 93 (91.20), 77 (68.40).

*N-(3-Cyano-4,5,6,7-tetrahydrobenzo[b]thiophen-2-yl)-N^\^-phenyl-2-(phenylhydrazono)malonamide* (**10**): *Method (A)*: To a cold solution (0-5 °C) of **4** (3.39 g, 0.01 mol), in ethanol (20 mL) containing sodium hydroxide (1.00 g) an equimolar amount of diazotized aniline was gradually added while stirring. The solid product formed upon cooling in an ice-bath was collected by filtration, washed with water and crystallized from 1,4-dioxane. *Method (B)*: To a solution of **9a** (3.96 g, 0.01 mol) in dimethylformamide (30 mL), aniline (0.93 g, 0.01 mol) was added. The reaction mixture was heated under reflux for 5 h. The solid product formed upon pouring onto ice/water mixture was collected by filtration, washed with water and crystallized from 1,4-dioxane. Pale orange crystals, m.p. 106-110 °C, yield: 3.41 g (77% in method **A** and **B**); Anal. For C_24_H_21_N_5_O_2_S (443.52), (% Calcd./Found): 64.99/64.62 (C), 4.77/4.36 (H), 15.79/15.57 (N), 7.23/7.67 (S); IR (*ν*, cm^−1^):3290-3209 (resonating OH, 3NH), 3063 (CH aromatic), 2934-2857 (CH_2_ cyclohexene), 2210 (CN), 1667, 1598, (2C=O) 1527,1443 (C=C); ^1^H-NMR (δ, ppm): 1.71-2.62 (m, 8H, cyclohexene 4CH_2_), 7.26-7.57 (m, 10H, 2C_6_H_5_), 10.14, 11.76, 13.35 (3s, 1H each, 3NH), 13.78 (s, 1H, resonating OH); MS *m/z* (%): 443 [M^+^] (3.50), 339 (18.10), 178 (100.00), 93 (91.20), 77 (68.40).

#### 3.2.4. Synthesis of Functionalized 4,5,6,7-Tetrahydrobenzo[*b*]thiophen-2-yl-3-cyano-, 3-Carboxylic Acid Ethyl Esters or 3-Carboxylic Acid Phenyl Amides **11a-d**, **12a-b** or **13a-b**

Compounds **11a-d** have been published earlier by our group [42].

##### 3.2.4.1. Synthesis of **12a-b**, **13a-b**

To a solution of compound **3** (2.92 g, 0.01 mol) or **4** (3.39 g, 0.01 mol) in 1,4-dioxane (25 mL) and dimethylformamide (10 mL) containing triethylamine (1.00 mL), either malononitrile (0.66 g, 0.01 mol) or ethyl cyanoacetate (1.13 g, 0.01 mol) was added. The reaction mixture, in each case, was heated under reflux for 5 h, then cooled and neutralized by pouring onto ice/water mixture containing few drops of hydrochloric acid. The solid product formed, in each case, was filtered off and crystallized from 1,4-dioxane/dimethylformamide mixture. 

*4,6-Diamino-1-(3-cyano-4,5,6,7-tetrahydrobenzo[b]thiophen-2-yl)-2-oxo-1,2-dihydropyridine-3-carboxylic acid ethyl ester* (**12a**): Brown crystals, m.p. 276-280 °C, yield: 2.36 g (66%); Anal. For C_17_H_18_N_4_O_3_S (358.41), (% Calcd./Found): 56.97/57.00 (C), 5.06/5.18 (H), 15.63/15.57 (N), 8.95/9.10 (S); IR (*ν*, cm^−1^): 3400-3188 (2NH_2_), 3083-3002 (CH aromatic), 2932-2852 (CH_3_, CH_2_), 2209 (CN), 1673, 1630 (2C=O), 1543, 1461 (C=C); ^1^H-NMR (δ, ppm): 1.12 (t, *J* = 7.65 Hz, 3H, ester CH_3_), 1.72-2.85 (m, 8H, cyclohexene 4CH_2_), 3.30-3.34 (s, 2H, NH_2_), 3.82 (s, 2H, NH_2_), 3.05 (q, *J* = 7.65 Hz, 2H, ester CH_2_), 7.92 (s, 1H, pyridine C5-H); MS *m/z* (%): 357 [M^+^–1] (12.37), 356 [M^+^–2] (18.51), 178 (100.00), 150 (99.10).

*4-Amino-1-(3-cyano-4,5,6,7-tetrahydrobenzo[b]thiophen-2-yl)-6-hydroxy-2-oxo-1,2-dihydropyridine-3-carboxylic acid ethyl ester* (**12b**): Pale yellow crystals, m.p. 268-270 °C, yield: 2.59 g (72%); Anal. For C_17_H_17_N_3_O_4_S (359.40), (% Calcd./Found): 56.81/57.01 (C), 4.77/4.40 (H), 11.69/11.82 (N), 8.92/9.20 (S); IR (*ν*, cm^−1^): 3740-3191 (OH, NH_2_), 3081 (CH aromatic), 2932-2852 (CH_3_, CH_2_), 2208 (CN), 1676, 1635 (2C=O), 1546, 1460 (C=C); ^1^H-NMR (δ, ppm): 1.14 (t, *J* = 7.50 Hz, 3H, ester CH_3_), 1.75-2.89 (m, 8H, cyclohexene 4CH_2_), 3.85 (s, 2H, NH_2_), 4.07 (q, *J* = 7.50 Hz, 2H, ester CH_2_), 6.90 (s, 1H, pyridine C5-H), 11.76 (s, 1H, OH) ; MS *m/z* (%): 360 [M^+^+1] (0.11), 359 [M^+^] (0.09), 178 (100.00), 150 (76.57).

*4,6-Diamino-1-(3-cyano-4,5,6,7-tetrahydrobenzo[b]thiophen-2-yl)-2-oxo-1,2-dihydropyridine-3-carboxylic acid phenylamide* (**13a**): Yellowish white crystals, m.p. 210-212 °C, yield: 2.84 g (70%); Anal. For C_21_H_19_N_5_O_2_S (405.47), (% Calcd./Found): 62.21/61.91 (C), 4.72/4.92 (H), 17.27/17.00 (N), 7.91/8.10 (S); IR (*ν*, cm^−1^): 3400-3282 (resonating OH, NH, 2NH_2_), 3081 (CH aromatic), 2932 (CH_2_ cyclohexene), 2213 (CN), 1663, 1600, (2C=O) 1544,1442 (C=C); ^1^H-NMR (δ, ppm): 1.72-2.94 (m, 8H, cyclohexene 4CH_2_), 3.44 (s, 2H, NH_2_), 3.64 (s, 2H, NH_2_), 6.92 (s, 1H, pyridine C5-H), 7.27-7.56 (m, 5H, C_6_H_5_), 10.18 (s, 1H, NH), 11.77 (s, 1H, resonating OH); MS *m/z* (%): 405 [M^+^], 407 [M^+^+2] (41.83), 405 [M^+^] (42.89), 54 (100.00).

*4-Amino-1-(3-cyano-4,5,6,7-tetrahydrobenzo[b]thiophen-2-yl)-6-hydroxy-2-oxo-1,2-dihydropyridine-3-carboxylic acid phenylamide* (**13b**): Pale yellow crystals, m.p. 135-138 °C, yield: 2.48 g (61%); Anal. For C_21_H_18_N_4_O_3_S (406.46), (% Calcd./Found): 62.05/61.72 (C), 4.46/4.72 (H), 13.78/13.40 (N), 7.89/8.21 (S); IR (*ν*, cm^−1^): 3426-3221 (OH, NH, NH_2_), 3082 (CH aromatic), 2932-2848 (CH_2_ cyclohexene), 2202 (CN), 1662, 1613, (2C=O) 1536,1441 (C=C); ^1^H-NMR (δ, ppm): 1.70-2.59 (m, 8H, cyclohexene 4CH_2_), 3.68 (s, 2H, NH_2_), 6.90 (s, 1H, pyridine C5-H), 7.03-7.61 (m, 5H, C_6_H_5_), 10.13-10.17 (s, 1H, NH), 11.75 (s, 1H, OH).

#### 3.2.5. Synthesis of Functionalized 4,5,6,7-Tetrahydrobenzo[*b*]thiophen-2-yl-5-phenylazo Pyridine Derivatives **14a-d** and **15a-b**

To a cold solution (0-5 °C) of **11a** (3.11 g, 0.01 mol), **11b** (3.12 g, 0.01 mol), **11c** (3.09 g, 0.01 mol), **11d** (3.11 g, 0.01 mol), **12a** (3.58 g, 0.01 mol) or **12b** (3.59 g, 0.01 mol), in ethanol (20 mL) containing sodium hydroxide (1.00 g) an equimolar amount of diazotized aniline was gradually added while stirring. The solid products formed upon cooling in an ice-bath were collected by filtration, washed with water and crystallized from 1,4-dioxane.

*4,6-Diamino-1-(3-cyano-4,5,6,7-tetrahydrobenzo[b]thiophen-2-yl)-2-oxo-5-phenylazo-1,2-dihydro-pyridine-3-carbonitrile* (**14a**): Orange crystals, m.p. 255-260 °C, yield: 3.45 g (83%); Anal. For C_21_H_17_N_7_OS (415.47), (% Calcd./Found): 60.71/60.41 (C), 4.12/4.30 (H), 23.60/23.21 (N), 7.72/8.11 (S); IR (*ν*, cm^−1^): 3446-3220 (2NH_2_), 3139-3067 (CH aromatic), 2930-2855 (CH_2 _cyclohexene), 2213, 2195 (2CN), 1679 (C=O), 1608, 1490 (C=C), 1545 (N=N); ^1^H-NMR (δ, ppm): 1.77-2.64 (m, 8H, cyclohexene 4CH_2_), 3.90, 4.10 (2s, 2H each, 2NH_2_), 7.15-7.69 (m, 5H, C_6_H_5_).

*4-Amino-1-(3-cyano-4,5,6,7-tetrahydrobenzo[b]thiophen-2-yl)-6-hydroxy-2-oxo-5-phenylazo-1,2-dihydro-pyridine-3-carbonitrile* (**14b**): Dark orange crystals, m.p. 230-235 °C, yield: 4.12 g (99%); Anal. For C_21_H_16_N_6_O_2_S (416.11), (% Calcd./Found): 60.56/60.72 (C), 3.87/3.74 (H), 20.18/19.94 (N), 7.70/8.00 (S); IR (*ν*, cm^−1^): 3444 (OH), 3358-3217 (NH_2_), 3064 (CH aromatic), 2928-2850 (CH_2_ cyclohexene), 2210, 2195 (2CN), 1679 (C=O), 1600, 1452 (C=C), 1539 (N=N); ^1^H-NMR (δ, ppm): 1.75-2.64 (m, 8H, cyclohexene 4CH_2_), 4.09 (s, 2H, NH_2_), 7.02-7.84 (m, 5H, C_6_H_5_), 12.65 (s, 1H, OH).

*1-(3-Cyano-4,5,6,7-tetrahydrobenzo[b]thiophen-2-yl)-4,6-dimethyl-2-oxo-5-phenylazo-1,2-dihydro-pyridine-3-carbonitrile* (**14c**): Orange crystals, m.p. 140-145 °C, yield: 2.60 g (63%); Anal. For C_23_H_19_N_5_OS (413.13), (% Calcd./Found): 66.81/66.43 (C), 4.63/4.42 (H), 16.94/16.60 (N), 7.75/8.10 (S); IR (*ν*, cm^−1^): 3100 (CH aromatic), 2933-2856 (CH_2_ cyclohexene), 2218, 2195 (2CN), 1671 (C=O), 1575, 1446 (C=C), 1541 (N=N); ^1^H-NMR (δ, ppm): 1.81, 2.16 (2s, 6H, 2CH_3_), 2.41-2.79 (m, 8H, cyclohexene 4CH_2_), 6.56-7.71 (m, 5H, C_6_H_5_); MS *m/z* (%): 413 [M^+^], 412 [M^+^–1] (5.90), 411 [M^+^–2] (23.50), 77 (41.20), 51 (100.00).

*1-(3-Cyano-4,5,6,7-tetrahydrobenzo[b]thiophen-2-yl)-6-hydroxy-4-methyl-2-oxo-5-phenylazo-1,2-dihydro-pyridine-3-carbonitrile* (**14d**): Pale brown crystals, m.p. 165-170 °C, yield: 3.11 g (75%); Anal. For C_22_H_17_N_5_O_2_S (415.11), (% Calcd./Found): 63.60/63.22 (C), 4.12/4.41 (H), 16.86/17.10 (N), 7.72/8.01 (S); IR (*ν*, cm^−1^): 3450-3219 (OH), 3083 (CH aromatic), 2933-2856 (CH_3_, CH_2 _cyclohexene), 2214, 2195 (2CN), 1685 (C=O), 1630, 1492 (C=C), 1546 (N=N); ^1^H-NMR (δ, ppm): 1.76 (s, 3H, CH_3_), 2.49-2.64 (m, 8H, cyclohexene 4CH_2_), 7.17-7.69 (m, 5H, C_6_H_5_), 11.88 (s, 1H, OH).

*4,6-Diamino-1-(3-cyano-4,5,6,7-tetrahydrobenzo[b]thiophen-2-yl)-2-oxo-5-phenylazo-1,2-dihydro-pyridine-3-carboxylic acid ethyl ester* (**15a**): Pale orange crystals, m.p. 217-222 °C, yield: 3.65 g (79%); Anal. For C_23_H_22_N_6_O_3_S (462.52), (% Calcd./Found): 59.73/60.10 (C), 4.79/4.40 (H), 18.17/18.43 (N), 6.93/6.60 (S); IR (ν, cm^−1^): 3348-3191 (2NH_2_), 3079 (CH aromatic), 2933-2851 (CH_3_, CH_2_), 2211 (CN), 1671, 1630 (2C=O), 1580, 1455 (C=C), 1530 (N=N); ^1^H-NMR (δ, ppm): 1.33 (t, *J* = 7.20 Hz, 3H, ester CH_3_), 1.75-2.58 (m, 8H, cyclohexene 4CH_2_), 4.30 (q, *J* = 7.20 Hz, 2H, ester CH_2_), 7.19-7.71 (m, 5H, C_6_H_5_), 3.19, 3.80 (2s, 2H each, 2NH_2_).

*4-Amino-1-(3-cyano-4,5,6,7-tetrahydrobenzo[b]thiophen-2-yl)-6-hydroxy-2-oxo-5-phenylazo-1,2-dihydro-pyridine-3-carboxylic acid ethyl ester* (**15b**): Pale orange crystals, m.p. 180-185 °C, yield: 3.16 g (78%); Anal. For C_23_H_21_N_5_O_4_S (463.51), (% Calcd./Found): 59.60/59.90 (C), 4.57/4.20 (H), 15.11/15.40 (N), 6.92/6.60 (S); IR (*ν*, cm^−1^): 3440-3202 (OH, NH_2_), 3079 (CH aromatic), 2932-2851 (CH aliphatic), 2211 (CN), 1675, 1640 (2C=O), 1590, 1455 (C=C), 1533 (N=N); ^1^H-NMR (δ, ppm): 1.32 (t, *J* = 6.90 Hz, 3H, ester CH_3_), 1.75-2.64 (m, 8H, cyclohexene 4CH_2_), 3.85 (s, 2H, NH_2_), 4.31 (q, *J* = 6.90 Hz, 2H, ester CH_2_), 7.22-7.74 (m, 5H, C_6_H_5_), 11.77 (s, 1H, OH).

#### 3.2.6. Synthesis of Functionalized 4,5,6,7-Tetrahydrobenzo[*b*]thiophen-2-yl-2-phenyl Azo Pyridine Derivatives **16a-b**

Compounds **16a-b** have been published earlier by our group [42].

#### 3.2.7. Synthesis of Functionalized 4,5,6,7-Tetrahydrobenzo[*b*]thiophen-2-yl-2-oxopyridine Derivatives **17a-b**

Compounds **17a-b** have been published earlier by our group [51].

#### 3.2.8. Synthesis of Functionalized 4,5,6,7-Tetrahydrobenzo[*b*]thiophen-2-yl-thiophene Derivatives **18a-b** and Thiazole Derivative **19**

Equimolar amounts of **4** (3.39 g, 0.01 mol) and phenyl isothiocyanate (1.35 g, 0.01 mol) in dimethylformamide (20 mL) and potassium hydroxide were stirred overnight. Ethyl chloroacetate (1.22 g, 0.01 mol), phenacyl bromide (1.99 g, 0.01 mol), or chloroacetone (0.92 g, 0.01 mol) were then added to the reaction mixture while stirring overnight. The solid products formed upon pouring onto ice/water mixture containing few drops of hydrochloric acid were collected by filtration and crystallized from 1,4-dioxane. 

*5-Ethoxycarbonyl-2,4-bis-phenylamino-thiophene-3-carboxylic acid (3-cyano-4,5,6,7-tetrahydrobenzo[b]-thiophen-2-yl)-amide* (**18a**): Pale brown crystals, m.p. 94-96 °C, yield: 4.88 g (90%); Anal. For C_29_H_26_N_4_O_3_S_2_ (542.67), (% Calcd./Found): 64.18/63.91 (C), 4.83/4.72 (H), 10.32/10.50 (N), 11.82/12.01 (S); IR (*ν*, cm^−1^): 3283-3197 (3NH), 3058 (CH aromatic), 2929-2854 (CH_3_, CH_2_), 2208 (CN), 1729, 1603, (2C=O) 1538,1499 (C=C); ^1^H-NMR (δ, ppm): 1.16 (t, *J* = 6.90 Hz, 3H, ester CH_3_), 1.72-2.85 (m, 8H, cyclohexene 4CH_2_), 4.08 (q, *J* = 6.90 Hz, 2H, ester CH_2_), 6.84-7.92 (m, 10H, 2C_6_H_5_), 9.82, 10.15, 11.51 (3s, 1H each, 3NH); MS *m/z* (%): 543 [M^+^+1] (22.07), 353 (100.00), 325 (56.14). 

*5-Benzoyl-2,4-bis-phenylamino-thiophene-3-carboxylic acid (3-cyano-4,5,6,7-tetrahydrobenzo[b]thio-phen-2-yl)-amide* (**18b**): Brown crystals, m.p. 78-82 °C, yield: 5.63 g (98%); Anal. For C_33_H_26_N_4_O_2_S_2_ (574.72), (% Calcd./Found): 68.97/68.62 (C), 4.56/4.83 (H), 9.75/10.01 (N), 11.16/11.40 (S); IR (*ν*, cm^−1^): 3276 (3NH), 3059 (CH aromatic), 2928-2853 (CH_2_ cyclohexene), 2207 (CN), 1644, 1548, (2C=O) 1496,1443 (C=C); ^1^H-NMR (δ, ppm): 1.71-2.85 (m, 8H, cyclohexene 4CH_2_), 6.96-7.98 (m, 15H, 3C_6_H_5_), 9.20, 10.15, 11.51 (3s, 1H each, 3NH); ^13^C NMR(δ, ppm): 22.8, 23.3, 23.8, 24.0 (4CH_2_), 92.6 (thiophene-C3), 114.8 (CN), 119.6, 126.3, 127.1, 127.4, 128.6, 128.8, 131.6, 131.9 147.2, 153.3, 157.3 (3C_6_H_5_, thiophene 3C), 129.3, 129.7 (thiophene-C5), 130.8, 131.0 (thiophene-C4), (thiophene-C2), 162.8, 165.9 (2C=O); MS *m/z* (%): 574 [M^+^] (12.97), 572 [M^+^–2] (20.01), 413 (100.00), 313 (58.64), 150 (15.81).

*N-(3-Cyano-4,5,6,7-tetrahydrobenzo[b]thiophen-2-yl)-2-(4-methyl-3-phenyl-3H-thiazol-2-ylidene)-N^\^-phenylmalonamide* (**19**): Reddish brown crystals, m.p. 88-90 °C, yield: 4.41 g (86%); Anal. For C_28_H_24_N_4_O_2_S_2_ (512.65), (% Calcd./Found): 65.60/65.51 (C), 4.72/4.60 (H), 10.93/11.21 (N), 12.51/12.20 (S); IR (*ν*, cm^−1^): 3415-3282 (2NH), 3062 (CH aromatic), 2926-2847 (CH_3_, CH_2_ cyclohexene), 2204 (CN), 1655, 1600, (2C=O) 1545,1491 (C=C); ^1^H-NMR (δ, ppm): 1.22 (s, 3H, CH_3_), 1.52-2.89 (m, 8H, cyclohexene 4CH_2_), 6.62 (s, 1H, thiazole C_5_), 6.93-7.95 (m, 10H, 2C_6_H_5_), 10.19, 11.48 (2s, 1H each, 2NH); ^13^C NMR(δ, ppm): 18.8 (CH_3_), 22.8, 23.3, 23.8, 24.0 (4CH_2_), 97.5 (thiophene-C3), 96.8, 104.5 (C=C), 115.9 (CN), 119.6, 125.6, 128.1, 128.7, 129.0, 129.3, 129.7, 132.7, 137.1, 140.0, 159.0 (2C_6_H_5_, thiophene, thiazole C), 161.3, 166.2 (2C=O); MS *m/z* (%): 512 [M^+^] (0.03), 351 (92.46), 323 (44.41), 77 (100.00).

#### 3.2.9. Synthesis of *5-Amino-4-cyano-3-hydroxy-thiophene-2-carboxylic acid (3-cyano-4,5,6,7-tetra-hydrobenzo[b]thiophen-2-yl)-amide* (**20**)

To a solution of **3** (2.92 g, 0.01 mol) in ethanol (25 mL) and dimethylformamide (5 mL) containing triethylamine (1.00 mL), malononitrile (0.66 g, 0.01 mol) was added followed by the addition of elemental sulfur (0.32 g, 0.01 mol). The reaction mixture was heated under reflux for 5 h, then cooled and neutralized by pouring onto ice/water mixture containing few drops of hydrochloric acid. The solid product formed was collected by filtration and crystallized from dimethylformamide. Brown crystals, m.p. 277-280 °C, yield: 3.27 g (95%); Anal. For C_15_H_12_N_4_O_2_S_2_ (344.41), (% Calcd./Found): 52.31/52.60 (C), 3.51/3.70 (H), 16.27/16.06 (N), 18.62/18.30 (S); IR (*ν*, cm^−1^): 3333-3196 (OH, NH, NH_2_), 3083 (CH aromatic), 2931-2852 (CH_2_ cyclohexene), 2250, 2209 (CN),1631 (C=O), 1546, 1459 (C=C); ^1^H-NMR (δ, ppm): 1.72-2.85 (m, 8H, cyclohexene 4CH_2_), 3.82 (s, 2H, NH_2_), 6.92 (s, 1H, NH), 11.79 (s, 1H, OH); ^13^C-NMR(δ, ppm): 23.7, 24.0, 24.3, 24.7 (4CH_2_), 93.3 (thiophene C-3), 114.6, 116.8 (2 CN), 126.8, 129.0, 130.6, 131.3, 133.8, 146.9 (two thiophene C), 165.0 (C=O); MS *m/z* (%): 344 [M^+^] (1.26), 178 (62.51), 150 (100.00).

#### 3.2.10. Synthesis of Functionalized 5-Oxopyrazole Derivatives **21a-b** and the Respective Hydrazono Dyes **22a-b**

To a solution of compound **3** (2.92 g, 0.01 mol) or **9a** (3.96 g, 0.01 mol) in 1,4-dioxane (25 mL) and dimethylformamide (10 mL), either hydrazine hydrate (0.50 g, 0.01 mol), or phenyl hydrazine (1.08 g, 0.01 mol) was added. The reaction mixture, in each case, was heated under reflux for 5 h. The solid product formed, in each case, upon pouring onto ice/water mixture was collected by filtration, and crystallized from 1,4-dioxane/dimethylformamide mixture.

*2-(5-Oxo-4,5-dihydro-1H-pyrazol-3-ylamino)-4,5,6,7-tetrahydrobenzo[b]thiophene-3-carbonitrile* (**21a**): Creamy crystals, m.p. 290-292 °C, yield: 2.52 g (97%); Anal. For C_12_H_12_N_4_OS (260.31), (% Calcd./Found): 55.37/55.60 (C), 4.65/4.32 (H), 21.52/21.22 (N), 12.32/11.94 (S); IR (*ν*, cm^−1^): 3431-3207 (2NH), 3087-3006 (CH aromatic), 2935-2857 (CH_2_ cyclohexene), 2211 (CN), 1680 (C=O), 1548, 1463 (C=C); ^1^H-NMR (δ, ppm): 1.76-2.59 (m, 8H, cyclohexene 4CH_2_), 3.85 (s, 2H, CH_2_), 6.95 (s, 1H, pyrazole NH), 11.77 (s, 1H, NH); MS *m/z* (%): 262 [M^+^+2] (0.51), 220 (4.15), 205 (8.39), 178 (100.00), 150 (75.36).

*2-(5-Oxo-1-phenyl-4,5-dihydro-1H-pyrazol-3-ylamino)-4,5,6,7-tetrahydrobenzo[b]thiophene-3-carbonitrile* (**21b**): Creamy crystals, m.p. 280-282 °C, yield: 2.66 g (79%); Anal. For C_18_H_16_N_4_OS (336.41), (% Calcd./Found): 64.26/64.00 (C), 4.79/4.88 (H), 16.65/16.30 (N), 9.53/9.80 (S); IR (*ν*, cm^−1^): 3431-3201 (NH), 3086-3006 (CH aromatic), 2935-2852 (CH_2_ cyclohexene), 2213 (CN), 1681 (C=O), 1546, 1463 (C=C); ^1^H-NMR (δ, ppm): 1.75-2.59 (m, 8H, cyclohexene 4CH_2_), 3.85 (s, 2H, CH_2_), 6.70-7.20 (m, 5H, C_6_H_5_), 11.77 (s, 1H, NH); MS *m/z* (%): 337 [M^+^+1] (4.49), 336 [M^+^] (4.25), 334 [M^+^–2] (4.82), 284 (43.09), 77 (100.00).

*2-[5-Oxo-4-(phenyl-hydrazono)-4,5-dihydro-1H-pyrazol-3-ylamino]-4,5,6,7-tetrahydrobenzo[b]-thiophene-3-carbonitrile* (**22a**): Orange crystals, m.p. 135-137 °C, yield: 3.21 g (88%); Anal. For C_18_H_16_N_6_OS (364.42), (% Calcd./Found): 59.32/59.00 (C), 4.43/4.33 (H), 23.06/22.80 (N), 8.80/9.10 (S); IR (*ν*, cm^−1^): 3343-3152 (resonating OH, 3NH), 3059 (CH aromatic), 2932-2848 (CH_2_ cyclohexene), 2208 (CN), 1665 (C=O), 1527,1450 (C=C); ^1^H-NMR (δ, ppm): 1.74-2.85 (m, 8H, cyclohexene 4CH_2_), 6.92-7.70 (m, 5H, C_6_H_5_), 8.83 (s, 1H, pyrazole NH), 10.73, 11.20 (2s, 1H each, 2NH), 12.58 (s, 1H, resonating OH); MS *m/z* (%): 366 [M^+^+2] (4.00), 365 [M^+^+1] (4.60), 178 (25.28), 150 (14.39), 92 (100.00), 77 (30.97).

*2-[5-Oxo-1-phenyl-4-(phenyl-hydrazono)-4,5-dihydro-1H-pyrazol-3-ylamino]-4,5,6,7-tetrahydro-benzo[b]thiophene-3-carbonitrile* (**22b**): Pale orange crystals, m.p. 106-110 °C, yield: 3.17 g (72%); Anal. For C_24_H_20_N_6_OS (440.52), (% Calcd./Found): 65.44/65.30 (C), 4.58/4.61 (H), 19.08/18.80 (N), 7.28/7.56 (S); IR (*ν*, cm^−1^): 3744-3339 (resonating OH, 2NH), 3054 (CH aromatic), 2931-2850 (CH_2_ cyclohexene), 2208 (CN), 1663 (C=O), 1528,1452 (C=C); ^1^H-NMR (δ, ppm): 1.73-2.94 (m, 8H, cyclohexene 4CH_2_), 6.22-7.65 (m, 10H, 2C_6_H_5_), 8.83, 12.50 (2s, 1H each, 2NH), 13.61 (s, 1H, resonating OH); MS *m/z* (%): 442 [M^+^+2] (0.48), 319 (18.95), 178 (43.94), 150 (37.51), 92 (100.00), 77 (97.99).

#### 3.2.11. Synthesis of Functionalized 4,5,6,7-Tetrahydrobenzo[*b*]thiophen-2-yl-oxazin-ylidine Azo Dyes **23a-b**

To a solution of **10** (4.43 g, 0.01 mol) in ethanol (25 mL) and dimethylformamide (5 mL) containing triethylamine (1.00 mL), either malononitrile (0.66 g, 0.01 mol) or ethyl cyanoacetate (1.13 g, 0.01 mol) was added. The reaction mixture, in each case, was heated under reflux for 5 h, then cooled and neutralized by pouring onto ice/water mixture containing few drops of hydrochloric acid. The solid product formed, in each case, was filtered off and crystallized from ethanol/dimethylformamide mixture. 

*2-[6-Amino-3-(3-cyano-4,5,6,7-tetrahydrobenzo[b]thiophen-2-yl)-4-imino-3,4-dihydro[1,3]-oxazin-2-ylidene]-N-phenyl-2-phenylazo-aceamide* (**23a**): Greenish brown crystals, m.p. 115-120 °C, yield: 4.43 g (87%); Anal. For C_27_H_23_N_7_O_2_S (509.58), (% Calcd./Found): 63.64/63.52 (C), 4.55/4.70 (H), 19.24/18.90 (N), 6.29/6.52 (S); IR (*ν*, cm^−1^): 3324-3208 (NH, NH_2_), 3050 (CH aromatic), 2932 (CH_2_ cyclohexene), 2205 (CN), 1600 (C=O) 1525,1449 (C=C); ^1^H-NMR (δ, ppm): 1.72-2.69 (m, 8H, cyclohexene 4CH_2_), 3.64 (s, 2H, NH_2_), 6.92 (s, 1H, oxazine C5), 7.26-7.57 (m, 10H, 2C_6_H_5_), 8.27, 10.14 (2s, 1H each, 2NH); MS *m/z* (%): 509 [M^+^] (15.97), 489 (14.39), 256 (48.14), 57 (100.00).

*2-[6-Amino-3-(3-cyano-4,5,6,7-tetrahydrobenzo[b]thiophen-2-yl)-4-oxo-3,4-dihydro[1,3]oxazin-2-ylidene]-N-phenyl-2-phenylazo-aceamide* (**23b**): Brown crystals, m.p. 100-105 °C, yield: 4.44 g (87%); Anal. For C_27_H_22_N_6_O_3_S (510.57), (% Calcd./Found): 63.52/63.24 (C), 4.34/4.41 (H), 16.46/16.10 (N), 6.28/6.52 (S); IR (*ν*, cm^−1^): 3318 (NH, NH_2_), 3060 (CH aromatic), 2930 (CH_2_ cyclohexene), 2207 (CN), 1662, 1600 (2C=O) 1523,1447 (C=C); ^1^H-NMR (δ, ppm): 1.72-2.61 (m, 8H, cyclohexene 4CH_2_), 3.64 (s, 2H, NH_2_), 6.92 (s, 1H, oxazine C5), 7.27-7.58 (m, 10H, 2C_6_H_5_), 10.14 (s, 1H, NH); MS *m/z* (%): 511 [M^+^+1] (20.89), 487 (26.56), 138 (46.17), 97 (69.73), 57 (100.00).

#### 3.2.12. Synthesis of Functionalized Pyrimidine Phenyl Hydrazone Dyes **24a-b**

Equimolar amounts of **9a** (3.96 g, 0.01 mol) or **10** (4.43 g, 0.01 mol) and phenyl isothiocyanate (1.35 g, 0.01 mol) in 1,4-dioxane (20 mL) containing triethylamine (1.0 mL) were heated under reflux for 5 h. After cooling, the reaction mixtures were acidified by hydrochloric acid and the crude products were precipitated, collected by filtration and crystallized from dimethylformamide.

*2-[4,6-Dioxo-3-phenyl-5-(phenylhydrazono)-2-thioxotetrahydropyrimidin-1-yl]-4,5,6,7-tetra-hydro-benzo[b]thiophene-3-carbonitrile* (**24a**): Brown crystals, m.p. 288-290 °C, yield: 3.16 g (65%); Anal. For C_25_H_19_N_5_O_2_S_2_ (485.58), (% Calcd./Found): 61.84/61.60 (C), 3.94/4.12 (H), 14.42/14.10 (N), 13.21/12.92 (S); IR (*ν*, cm^−1^): 3447-3343 (resonating OH, NH), 3046 (CH aromatic), 2932-2853 (CH_2_ cyclohexene), 2206 (CN), 1665, 1620 (2C=O), 1527, 1451 (C=C); ^1^H-NMR (δ, ppm): 1.75-2.85 (m, 8H, cyclohexene 4CH_2_), 7.19-7.72 (m, 10H, 2C_6_H_5_), 12.58 (s, 1H, NH), 13.62 (s, 1H, resonating OH); MS *m/z* (%): 485 [M^+^], 484 [M^+^–1] (9.69), 436 (9.01), 178 (21.68), 150 (26.20), 92 (100.00), 77 (52.53).

*2-[6-Oxo-3-phenyl-5-(phenylhydrazono)-4-phenylimino-2-thioxotetrahydropyrimidin-1-yl]-4,5,6,7-tetrahydrobenzo[b]thiophene-3-carbonitrile* (**24b**): Dark green crystals, m.p. 68-70 °C, yield: 3.53 g (63%); Anal. For C_31_H_24_N_6_OS_2_ (560.69), (% Calcd./Found): 66.41/66.10 (C), 4.31/4.31 (H), 14.99/14.63 (N), 11.44/11.51 (S); IR (*ν*, cm^−1^): 3212-3119 (resonating OH, NH), 3047 (CH aromatic), 2930 (CH_2_ cyclohexene), 2206 (CN), 1664 (C=O) 1594,1443 (C=C), 1529 (=N-NH), 1326, 1290 (C=S); ^1^H-NMR (δ, ppm): 1.73-2.85 (m, 8H, cyclohexene 4CH_2_), 7.14-7.65 (m, 15H, 3C_6_H_5_), 11.03 (s, 1H, NH), 13.36 (s, 1H, resonating OH); MS *m/z* (%): 560 [M^+^], 561 [M^+^+1] (35.20), 559 [M^+^–1] (29.13), 150 (86.53), 106 (100.00), 93 (70.49).

### 3.3. Spectral Characterization, Colour Assessment and Dyeing Properties

#### 3.3.1. Dyeing Procedure

Unless otherwise indicated, dyeing was performed using a solution containing 5% dye (based on weight of sample), 2 g/L dispersing agent and ammonium persulphate at 120 °C for 45 min. A material to liquor ratio 1:20 was used. The dye solution was adjusted at Ph = 4.5-5 using acetic acid. After the end of dyeing time, the fabric sample was washed in a solution containing 5 g/L detergent for several times until a clear solution was obtained. Finally the fabric sample was rinsed with water and dried at ambient conditions. The colour of the dyes on cellulose triacetate, nylon 66 and polyester fibers are indicated [48] (Table 1).

#### 3.3.2. Colour Strength

Colour strength of the dyed samples expressed as (K/S) was measured at λ_max_ = 400 nm (Table 1).

#### 3.3.3. Fastness Properties

The colour fastness to washing, rubbing (dry and wet crocking) and perspiration was determined according to the standard method [49]. Data are indicated in Table 1.

##### 3.3.3.1. Colour Fastness to Washing

The composite specimens were sewed between two pieces of bleached cotton fabric and then immersed into an aqueous solution containing 5g/L soap non-ionic detergents at liquor ratio 50:1 and 2g/L sodium carbonate. The bath was thermostatically adjusted to 90 °C for 30 min; then samples were removed, rinsed twice with occasional hand squeezing, then dried. Evaluation of the wash fastness was established using the Grey-scale for colour change (Table 1).

##### 3.3.3.2. Colour Fastness to Rubbing

The test is designed for determining the degree of colour, which may transfer from the surface of the coloured fabric to another surface, by rubbing.

*Dry crocking test*: The test specimen was placed flat on the base of the crock-meter. A white testing cloth was mounted. The covered finger was lowered on to the test specimen and caused to slide 20 times back and forth by making ten complete turns at a rate of one turn/sec. The white test sample was then removed for evaluation using the Grey-scale for staining.

*Wet crocking test*: The white test sample was thoroughly wetted out in water to a 65% and then picked up. The procedure was run as above. The white test samples were air dried before evaluation.

##### 3.3.3.3. Colour Fastness to Perspiration

Two artificial perspiration solutions were prepared according to the following: 

*Acidic solution*: L-Histidine monohydrochloride monohydrate (0.5 g), sodium chloride (5 g) and sodium dihydrogen phosphate-1-hydrate (2.2 g) were dissolved in one liter distilled water. The pH was adjusted to 5.5 by 0.1N sodium hydroxide solution.

*Alkaline solution*: L-Histidine monohydrochloride monohydrate (0.5 g), sodium chloride (5 g) and di-sodium hydrogen phosphate-2-hydrate (2.5 g) were dissolved in one liter distilled water. The pH was adjusted to 8 by 0.1N sodium hydroxide solution.

The coloured specimen was sewed between two pieces of bleached cotton specimen. The composite sample was then immersed for 30 min. in the acidic perspiration solution at 37 °C (±2) with occasional agitation and squeezing to insure complete wetting. The test specimen was placed between two plastic plates under a force of about 5 Kg. The plates containing the composite specimens were left for about 6-8 hours. The same experiment was followed with another composite sample using the alkaline perspiration solution. The effect on the colour of the test specimen was expressed and defined by reference to Grey-scale for color change.

##### 3.3.3.4. Colour Fastness to Light

The light fastness test was assessed in accordance with test method (ISO 105-A03). Using Mercury-Tungsten lamp, (continuous light) for 40 hours. The effect on the colour of the test samples was expressed and defined by reference to Grey-scale for colour change.

### 3.4. Biology

#### 3.4.1. Antimicrobial Activity of the Synthesized Dyed and Dye Intermediate

Microorganisms used were obtained from Microbial Chemistry Department, National Research Center, Cairo, Egypt. For the *in vitro* antimicrobial activity evaluation, microorganism suspensions were prepared to contain approximately 108 cuf/mL and the plates were inoculated. A stock solution of each of the synthesized compounds (1.0 mg/mL) in DMSO was prepared and graded dilutions of the tested compounds were incorporated in a cavity (depth 3 mm, diameter 4 mm) made in the center of the Petri dish (nutrient agar for bacteria and Sabouraud *vs.* dextrose agar medium for fungi). The plates were incubated in duplicates for 24 h at 37 °C (for bacteria) and at 30 °C (for fungi). A positive control using only inoculation and a negative control using only DMSO in the cavity were carried out. The results of antimicrobial screening of the synthesized and standard antibiotics are given in Table 2.

#### 3.4.2. Antimicrobial Activity of the Dyed Fabrics

##### 3.4.2.1. Media Used

Nutrient broth/agar medium: contains (g/L), (5) peptone, (3) beef extract. For solid medium 15 g/L agar was added. Malt broth/agar medium: contains (g/L), (5) peptone, (24) malt extract. For solid medium 15 g/L agar was added. 2.7.3. Growth conditions an inoculum of each bacterial strain was suspended in 25 mL of nutrient broth medium and shaken for 24 h at 37 °C. For yeast, malt broth was inoculated with test organism and incubated at 28 °C for 24 h.

##### 3.4.2.2. Antimicrobial Activity Test

Disc diffusion method with some modifications was used for screening the nylon 66, acetate and polyester fabric samples for antimicrobial activity [52]. Nutrient agar (for bacteria) or malt agar (for yeast) plates were inoculated with 0.1 mL of an appropriate dilution of the tested culture. Test fabric samples (1 cm diameter) were placed on the surface of the inoculated plates. The plates were incubated at the appropriate temperature for 24 h. Diameter of inhibition zone (mm) including the disc diameter was measured for each treatment. The result of antimicrobial activity of the tested dyed fabric are given in Table 3.

## 4. Conclusions

Our synthetic strategy provides a simple protocol for producing dyes and dye precursors based on conjugate enaminones and/or enaminonitriles with potent dyeing and/or antimicrobial finishing capabilities. The novel compounds could be lead for the development of new functional materials with special finish properties for textile fabrics. Moreover, the results of the present study may point that the novel products could be useful as synthetic precursors for azo- and azomethine ligands or polymethine dyes which may be suitable for both electronic and optical applications. It is clear that fabrics dyed or treated with the synthesized novel systems exhibit significant antimicrobial activity. This is due to the inherent antimicrobial character of the dyes or dye precursors. The presence of an oxo moiety which could be responsible to complex or bind with nucleophilic amino acids in proteins leads to inactivation of the protein and loss of function. Moreover, formation of H-bonds with water molecules enable these compounds to more readily form positive ions, thereby inhibition of microbial growth by adsorption onto bacterial surface. As a consequence, we can conclude that the newly synthesized systems could be accepted as promising to develop new antibacterial compounds. 
